# Microbial metabolisms in an abyssal ferromanganese crust from the Takuyo-Daigo Seamount as revealed by metagenomics

**DOI:** 10.1371/journal.pone.0224888

**Published:** 2019-11-08

**Authors:** Shingo Kato, Miho Hirai, Moriya Ohkuma, Katsuhiko Suzuki

**Affiliations:** 1 Submarine Resources Research Center, Japan Agency for Marine-Earth Science and Technology (JAMSTEC), Yokosuka, Kanagawa, Japan; 2 Japan Collection of Microorganisms (JCM), RIKEN BioResource Research Center, Tsukuba, Ibaraki, Japan; 3 Research and Development Center for Marine Biosciences, JAMSTEC, Yokosuka, Kanagawa, Japan; University of Trento, ITALY

## Abstract

Rocky outcrops covered with thick Fe and Mn oxide coatings, which are known as ferromanganese (Fe-Mn) crusts, are commonly found on slopes of aged seamounts in bathyal and abyssal zones. Although the presence of diverse microorganisms on these Fe-Mn crusts has been reported, little is known about their metabolism. Here, we report the metabolic potential of the microbial community in an abyssal crust collected in the Takuyo-Daigo Seamount, in the north-western Pacific. We performed shotgun metagenomic sequencing of the Fe-Mn crust, and detected putative genes involved in dissolution and precipitation of Fe and Mn, nitrification, sulfur oxidation, carbon fixation, and decomposition of organics in the metagenome. In addition, four metagenome-assembled genomes (MAGs) of abundant members in the microbial community were recovered from the metagenome. The MAGs were affiliated with *Thaumarchaeota*, *Alphaproteobacteria*, and *Gammaproteobacteria*, and were distantly related to previously reported genomes/MAGs of cultured and uncultured species. Putative genes involved in the above reactions were also found in the crust MAGs. Our results suggest that crust microbial communities play a role in biogeochemical cycling of C, N, S, Fe, and Mn, and imply that they contribute to the growth of Fe-Mn crusts.

## Introduction

Ferromanganese (Fe-Mn) oxide coatings, such as Fe-Mn crusts and nodules, have been found in the pelagic deep ocean. Fe-Mn crusts generally cover rocky outcrops on slopes of aged and volcanically-inactive seamounts in bathyal and abyssal zones [1–3]. In some cases, the thickness of the crust is over 10 cm, which corresponds to a passage time of over ten million years (Myr) based on their extremely slow growth rate (1–10 mm/Myr) [1–4]. Fe-Mn oxides adsorb dissolved metallic ions from seawater [5, 6], and therefore, they are referred to as the “chemical scavengers of the sea” [7]. In fact, the deep-sea Fe-Mn coatings show ten times or higher concentrations of various elements such as cobalt (Co), molybdenum (Mo), tungsten (W), nickel (Ni), copper (Co), and zinc (Zn), in addition to Fe and Mn, than those in the seawater and even in Earth’s crustal rocks [8]. Many of these elements are essential for the growth of microbial life.

Despite the limitation of organic input from the ocean surface, the pelagic deep-sea Fe-Mn coatings harbor diverse and abundant microorganisms [9–13]. Indeed, the number of microbial cells on the surface is estimated to be 10^6^–10^8^ cells/g by microscopic direct counting or quantitative PCR [9, 11, 13, 14]. Considering that there are microorganisms contributing to the growth and dissolution of iron- and manganese-minerals [15–17], the microbial activities thriving in the Fe-Mn coatings are potentially involved in elemental cycling between the coatings and seawater. Previous 16S rRNA gene analyses have indicated that the microbial community structures of the Fe-Mn coatings are distinguishable from those of the surrounding seawater and sediments [10–14, 18], suggesting that a unique ecosystem develops in the Fe-Mn coatings. As-yet-uncultured lineages affiliated with *Gammaproteobacteria*, *Alphaproteobacteria*, and *Thaumarchaeota* are dominant in the microbial communities of the Fe-Mn coatings [9–14, 18–20]. In particular, 16S rRNA genes related to Marine Group I (MGI) [21] of the *Thaumarchaeota*, which include ammonia-oxidizing autotrophs such as *Nitrosopumilus* spp. [22], have been detected as a major lineage in the Fe-Mn coatings. The *amoA* genes related to the MGI ammonia oxidizers have been also detected [11]. This gene encodes a subunit of ammonia monooxygenase, a key enzyme for ammonia oxidation. Thus, ammonia likely serves as one of the main energy sources to sustain the microbial ecosystem in the Fe-Mn coatings. However, the metabolic functions of the as-yet-uncultured microorganisms present in the deep-sea Fe-Mn coatings are still largely unknown due to a lack of cultured representatives and even whole-genome sequences. Therefore, it remains unclear whether and how these microorganisms contribute to biogeochemical cycling between the Fe-Mn coatings and seawater, and to the formation of Fe-Mn coatings.

Here we report the metabolic potential of microorganisms in an abyssal Fe-Mn crust, as revealed by metagenomics. The Fe-Mn crust was collected at a water depth of 5520 m in the Takuyo-Daigo Seamount [13], where the availability of organic matter derived from the ocean surface is limited. This seamount is in the north-western Pacific on one of the oldest oceanic plates (> 150 Myr old [23, 24]). Fe-Mn crusts have been found covering the slope of most of the seamount, from the top at a water depth of 800 m to the bottom at a water depth of up to 6000 m [2]. Previous 16S rRNA gene analyses have indicated that diverse and abundant microorganisms are present throughout the water depth [11, 13, 14]. In the present study, we found putative genes involved in biogeochemical cycling of carbon (C), nitrogen (N), Fe, and Mn in the metagenome. Furthermore, we reconstructed metagenome-assembled genomes (MAGs) of as-yet-uncultured lineages of *Gammaproteobacteria*, *Alphaproteobacteria*, and *Thaumarchaeota*, which were detected as dominant members in the metagenome of the Fe-Mn coating. Although metagenomics of deep seawater [25–28], oligotrophic deep-sea surface sediments [29, 30], and seafloor basalts without Fe-Mn coatings [31] have been reported, so far there are no reports of metagenomics of deep-sea Fe-Mn coatings. Our results provide novel insights into the microbial contribution to the geochemical cycling and formation of the deep-sea Fe-Mn coatings.

## Materials and methods

### Field sampling

The sampling location and procedures have been previously described [13]. In brief, the Fe-Mn crust (the sample name, 680R2) was collected at a water depth of 5520 m at the Takuyo-Daigo Seamount during the KR16-E01 cruise (January 2016) with the research vessel (R/V) *Kairei* (JAMSTEC, Yokosuka, Kanagawa, Japan) and the remotely operated vehicle (ROV) *Kaiko Mk-IV* (JAMSTEC). Using the manipulators of the ROV on the seafloor, we transferred the crust sample into a sealable aluminum box to avoid contamination from surface seawater or sediments stirred up during the operation. On board, we subsampled the surface pieces of the Fe-Mn crust using sterile hammers and chisels. These pieces were stored at −80°C until DNA extraction.

### Metagenomics

DNA was extracted from the surface pieces (0.5 g; subsample ID, MnT55a) of the Fe-Mn crust using a FastDNA SPIN kit for soil (MP Biomedicals, Santa Ana, CA, USA) with a FastPrep instrument (MP Biomedicals) as previously described [13]. An aliquot of the extracted DNA has been previously used for 16S rRNA gene analysis [13]. Another aliquot was used for shotgun metagenomic sequencing in the present study. Library construction using a KAPA Hyper Prep Kit (KAPA Biosystems, Wilmington, MA, USA) and sequencing on an Illumina MiSeq platform (300 bp pair-end) were performed as previously described [32]. Sequence analysis was conducted as previously reported [33] with several modifications. For trimming and filtering of the reads, we used CLC Genomics Workbench version 9.5.3 (QIAGEN Aarhus A/S) with the default settings. The treated reads were assembled using SPAdes version 3.9.0 [34] with the following parameters: -k 55, 77, 99. Protein-coding DNA sequences (CDSs) in contigs (400 bp or longer) were predicted using Prodigal version 2.6.3 [35]. The treated reads were mapped to the contigs using BBmap version 36.49 (https://sourceforge.net/projects/bbmap) to calculate read coverage. Functional characterization of the CDSs was performed using the Kyoto Encyclopedia of Genes and Genomes (KEGG) pathway tool [36] with GhostKOALA [37]. Homology searching of targeted CDSs against a non-redundant (nr) database in NCBI was performed using BLASTp version 2.5.0+ [38] with the default parameters. Using the search results of the top 100 hits, CDSs were taxonomically classified using MEGAN community edition version 6.6.7 [39] with default settings using the lowest common ancestor (LCA) algorithm [40]. We used phyloFlash version 3.0 [41] to determine the abundance of 16S rRNA genes in the metagenome and their taxonomy against the SILVA database release 128. The prediction of metabolic pathways was performed using the KEGG pathway tool with BlastKOALA [37]. CDSs for glycoside hydrolase (GH) families with the CAZy classification [42] were determined using dbCAN2 [43] and HMMER version 3.1 [44] with the thresholds of *E*-value <1e–15 and coverage >0.35.

The contigs and mapping data were used for binning using Metabat version 0.32.4 with the default parameters [45]. The binned contigs were visualized for GC content vs. coverage using gbtools version 2.4.5 [46] and manually curated. The reads mapped to contigs of each curated bin were then re-assembled using SPAdes with the following parameter (-k 99, 127) to improve assembly. The reassembled contigs were binned using Metabat and curated using gbtools, which resulted in four metagenome-assembled genomes (MAGs). The sequences of the MAGs were annotated using Prokka version 1.11 [47]. Enveomics version 1.0 was used to calculate amino acid identities (AAIs) using the default parameters [48]. Completeness and contamination levels for the MAGs were calculated by counting conserved single-copy marker genes using CheckM version 1.0.7 [49].

### Phylogenetic analysis

Amino acid sequences were aligned using MUSCLE version 3.8.31 with the default parameters [50]. The alignment was trimmed using trimAl version 1.2rev59 with the option ‘-automated1’ [51]. Maximum likelihood trees were constructed using RAxML version 8.2.9 [52] with the GTRGAMMA model for nucleotide sequences and with the PROTGAMMALG model for amino acid sequences.

### Accession numbers

The raw sequence data obtained in this study have been deposited into the DNA Data Bank of Japan (DDBJ) under the accession number DRA006451 for shotgun metagenomic sequences. The sequence and annotation data of the MAGs have been deposited under the accession numbers BFAR01000001–BFAR01000180, BFAS01000001–BFAS01000378, BFAT01000001–BFAT01000343, BFAU01000001–BFAU01000244, for MnTg01 to MnTg04, respectively. Nucleotide sequences of all contigs assembled from the metagenome, amino acid and nucleotide sequences of CDSs in the contigs, and the alignment dataset used for the phylogenetic tree construction are available in FigShare (https://doi.org/10.6084/m9.figshare.5857878.v2).

## Results

To address the metabolic potential of microorganisms in the Fe-Mn crust, we performed shotgun metagenomic sequencing. This produced 8.4 million high-quality trimmed reads (total 2.18 Gb) with an average of 260 bases. The sequence assembly contained 754,038 contigs comprising 400 bases or longer, 63,795 contigs comprising 1 kb or longer, and 668 contigs comprising 10 kb or longer. A total of 1,008,143 protein-coding DNA sequences (CDSs) was obtained from all contigs.

### Membership of the crust community metagenome

**Fig 1:**
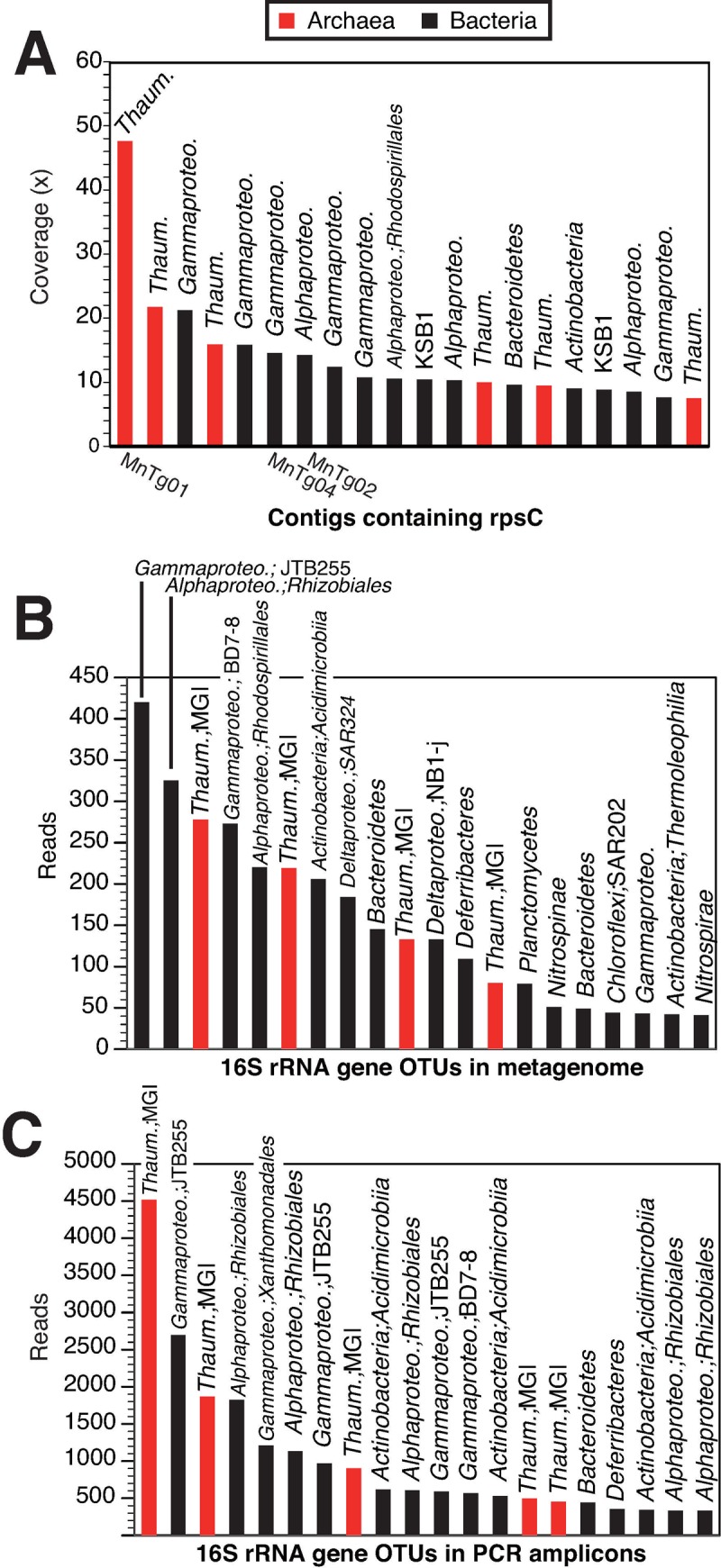
Rank abundant plot for phylogenetic marker genes. (A) Read coverage of contigs containing CDSs for RpsC, (B) read counts of 16S rRNA gene operational taxonomic units (OTUs) in the metagenome, and (C) read counts of 16S rRNA gene OTUs in the PCR amplicon obtained in the previous study [13]. Taxonomic affiliations for the CDSs for RpsC and 16S rRNA genes are indicated on the bars. The IDs of metagenome-assembled genomes (MAGs) are shown for the contigs containing CDSs identical to those of the MAGs. Red and black bars indicate archaeal and bacterial CDSs, respectively.

To assess the relative abundance of each microorganism in the metagenome, read coverages for contigs containing a CDS of the ribosomal protein S3 gene (*rpsC*), a single-copy marker gene that was used for such analysis (e.g., [53]), were analyzed. A rank abundant plot of the top 20 contigs showing higher read coverages is shown in Fig 1A. The phylogeny of the RpsC (S1 Fig) from the top 20 taxonomic groups was generally consistent with the taxonomic affiliation determined by LCA analysis. The top 20 results indicated that members of the *Thaumarchaeota*, *Gammaproteobacteria*, and *Alphaproteobacteria* were abundant in the metagenome. Particularly, the RpsC CDSs of *Thaumarchaeota* were classified into the clade of ammonia-oxidizing archaea (AOA), including *Nitrosomarinus* spp., *Nitrosopelagicus* spp., and *Nitrosopumilus* spp. (S1 Fig). Members of *Actinobacteria*, *Bacteroidetes*, and the candidate phylum KSB1 were also present in the top 20. The top 20 read counts of 16S rRNA genes in the metagenome and those in the previous 16S rRNA gene amplicon analysis [13] contained members of the above taxonomic groups, in addition to members of *Deltaproteobacteria*, *Nitrospirae*, *Nitrospinae*, and *Calditrichaeota* (Fig 1B and 1C). Discrepancies in the order of members observed among the results (metagenomics vs. PCR amplicon analysis, and *rpsC* vs. 16S rRNA genes) (Fig 1) were probably due to differences in the copy number of each gene and/or the efficiency of the PCR amplification.

### Metabolic potential of the crust community

**Fig 2:**
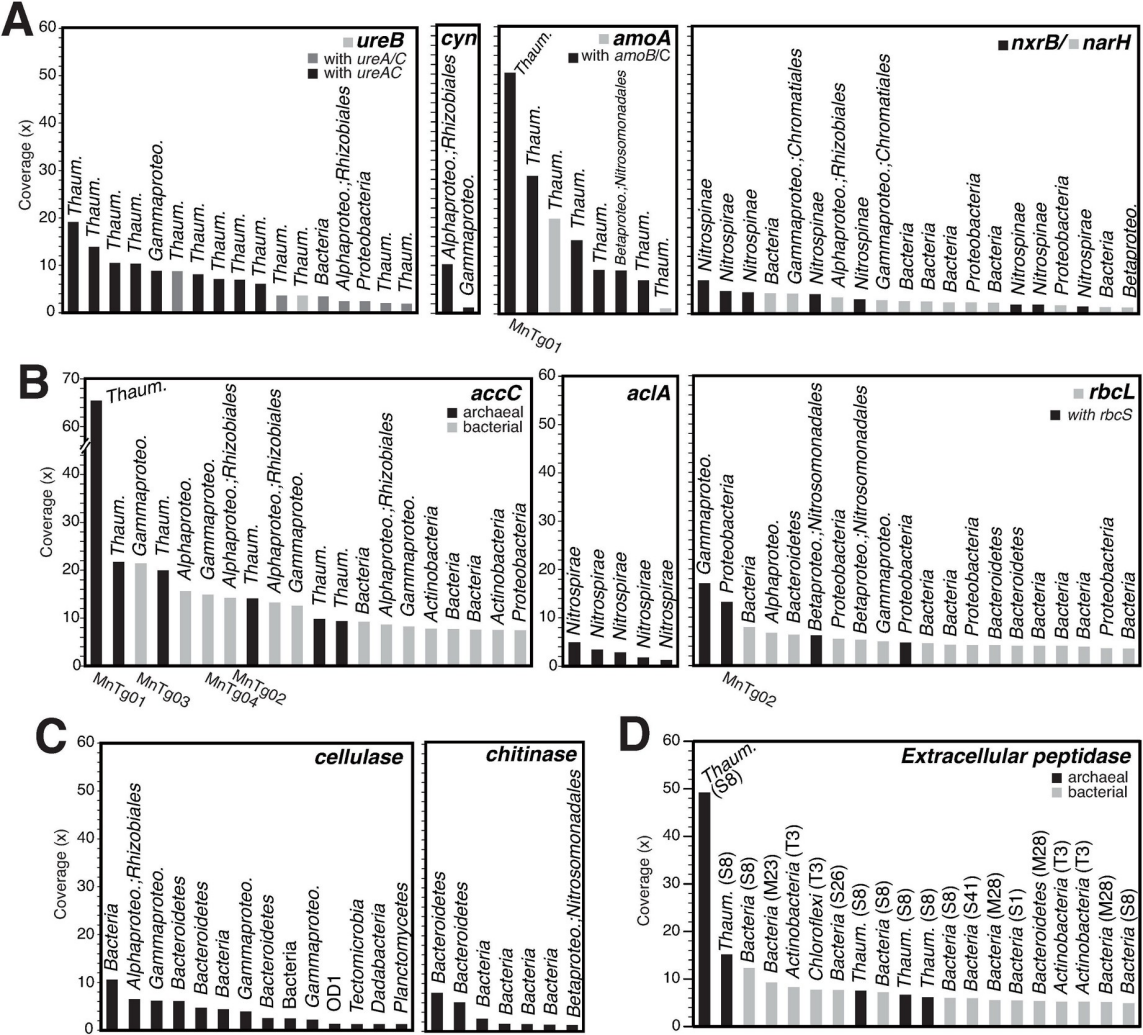
Read-coverage-based rank abundant plot of contigs containing CDSs potentially involved in carbon and nitrogen cycling. (A) CDSs for UreB, Cyn, AmoA, and NxrB/NarH involved in nitrification. (B) CDSs for AccC, AclA, and RbcL for key enzymes in carbon fixation pathways. (C) CDSs for cellulase and chitinase. (D) CDSs for extracellular peptidases. Peptidase family names for S, T, and M are indicated in parentheses. Taxonomic affiliations for the CDSs are indicated on the bars. The IDs of metagenome-assembled genomes (MAGs) are shown for the contigs containing CDSs identical to those of the MAGs.

Nitrification is an important metabolic process for energy acquisition in oligotrophic deep-sea environments, including the surface of Fe-Mn crusts, as suggested previously based on 16S rRNA and *amoA* gene analysis [11, 13, 14]. To assess the nitrification potential of the crust community, we analyzed CDSs for ammonia monooxygenase (Amo) [Enzyme Commission (EC) numbers; 1.14.99.39], cyanase (Cyn) [EC 4.2.1.104], urease (Ure) [EC 3.5.1.5], and nitrite oxidoreductase (Nxr) [EC 1.7.5.1] (Fig 2A), which are involved in NH_3_ oxidation to NO_2_^-^, cyanate and urea degradation to NH_3_ and CO_2_, and NO_2_^-^ oxidation to NO_3_^-^, respectively. Over ten thaumarchaeotic CDSs for UreB with the other subunits UreA and/or UreC were recovered from the metagenome, in addition to those affiliated with *Gammaproteobacteria* and *Alphaproteobacteria*. Only two alphaproteobacterial and gammaproteobacterial CDSs for Cyn were recovered. Seven thaumarchaeotic CDSs for AmoA with the other subunits AmoB and/or AmoC, and those affiliated with *Nitrosomonadales*, which include ammonia-oxidizing bacteria (AOB) such as *Nitrosomonas* spp. and *Nitrosospira* spp., were recovered. No CDSs for Amo related to *Nitrospira* were detected, which contain complete ammonia oxidizers (comammox), as recently reported [54, 55]. Because it was difficult to distinguish between Nxr and nitrate reductase (Nar) based only on homology searching, we constructed a phylogenetic tree for NarH/NxrB (S2 Fig), for which the subunit of Nar/Nxr has been used for functional and phylogenetic analysis [56]. Several Nar/Nxr CDSs in the metagenome were likely Nxr and were closely related to those encoded in the genomes of nitrite-oxidizing bacteria (NOB), such as *Nitrospira* spp. and *Nitrospina* spp. These AOA, AOB, and NOB are known to be autotrophs that fix inorganic carbon via the 3-hydroxypropionate/4-hydroxybutyrate (3HP/4HB) cycle, Calvin-Benson-Bassham (CBB) cycle, and reductive tricarboxylic acid cycle [57]. Indeed, CDSs for subunits of the key enzymes in these carbon fixation pathways, i.e., acetyl-CoA/propionyl-CoA carboxylase (Acc), ATP-citrate lyase (Acl), and ribulose-bisphosphate carboxylase (Rbc, or RubisCO), were recovered (Fig 2B). The bacterial CDSs for Acc were likely involved in fatty acid synthesis/degradation, rather than in carbon fixation.

Another potential energy source for the crust community is particulate organic carbon (POC) sinking from the ocean surface. We detected CDSs for cellulase [EC 3.2.1.4] and chitinase [EC 3.2.1.14] in the metagenome (Fig 2C), which were affiliated with some taxa, such as *Bacteroidetes*, *Gammaproteobacteria*, and *Alphaproteobacteria*. In addition, many CDSs for GH families were detected (S3 Fig). Especially, GH3 including glucosidase [EC 3.2.1.21], GH15 including glucoamylase [EC 3.2.1.3], GH23 including lysozyme type G [EC 3.2.1.17], GH103 including peptidoglycan lytic transglycosylase [EC 3.2.1.-], and GH130 including mannosylglucose phosphorylase [EC 2.4.1.281] accounted for over 50 CDSs of each GH family. We also detected CDSs for putative extracellular serine peptidase (peptidase families S1, S8, S26, and S41), metallopeptidase (M23 and M28), and threonine peptidase (T3) in the metagenome (Fig 2D), which were affiliated with some taxa, such as *Thaumarchaeota*, *Actinobacteria*, *Chloroflexi*, and *Bacteroidetes*. The above enzymes might be used to decompose polysaccharides and protein debris of POC.

Simple organic carbon compounds such as pyruvate, acetaldehyde, and formaldehyde, which can be produced by abiotic decomposition of POC by Mn oxides [58], can be also used by crust microorganisms. Indeed, CDSs for formaldehyde dehydrogenase catalyzing formate production from formaldehyde [EC 1.2.1.46] were detected in several contigs, although the coverages were relatively low (1×) (S4 Fig), in addition to CDSs for the utilization of pyruvate (such as pyruvate formate-lyase [EC 2.3.1.54], 2-oxoacid oxidoreductase [EC 1.2.7.11]), acetaldehyde (such as aldehyde dehydrogenase (ALDH) [EC 1.2.1.3] and aldehyde reductase [EC 1.1.1.1]). The above CDSs might be involved in organic carbon decomposition to generate ATP and reduce electron carriers such as NADH and quinol, and not only in assimilation of organic carbon. Furthermore, we found CDSs for lactate dehydrogenase (LDH) [EC 1.1.1.27] affiliated with *Planctomycetes* and *Thaumarchaeota* (S4 Fig). These CDSs for LDH and ALDH could be involved in lactate- and acetate-producing fermentation (resulting in a pH decrease) and/or lactate and acetate degradation.

**Fig 3:**
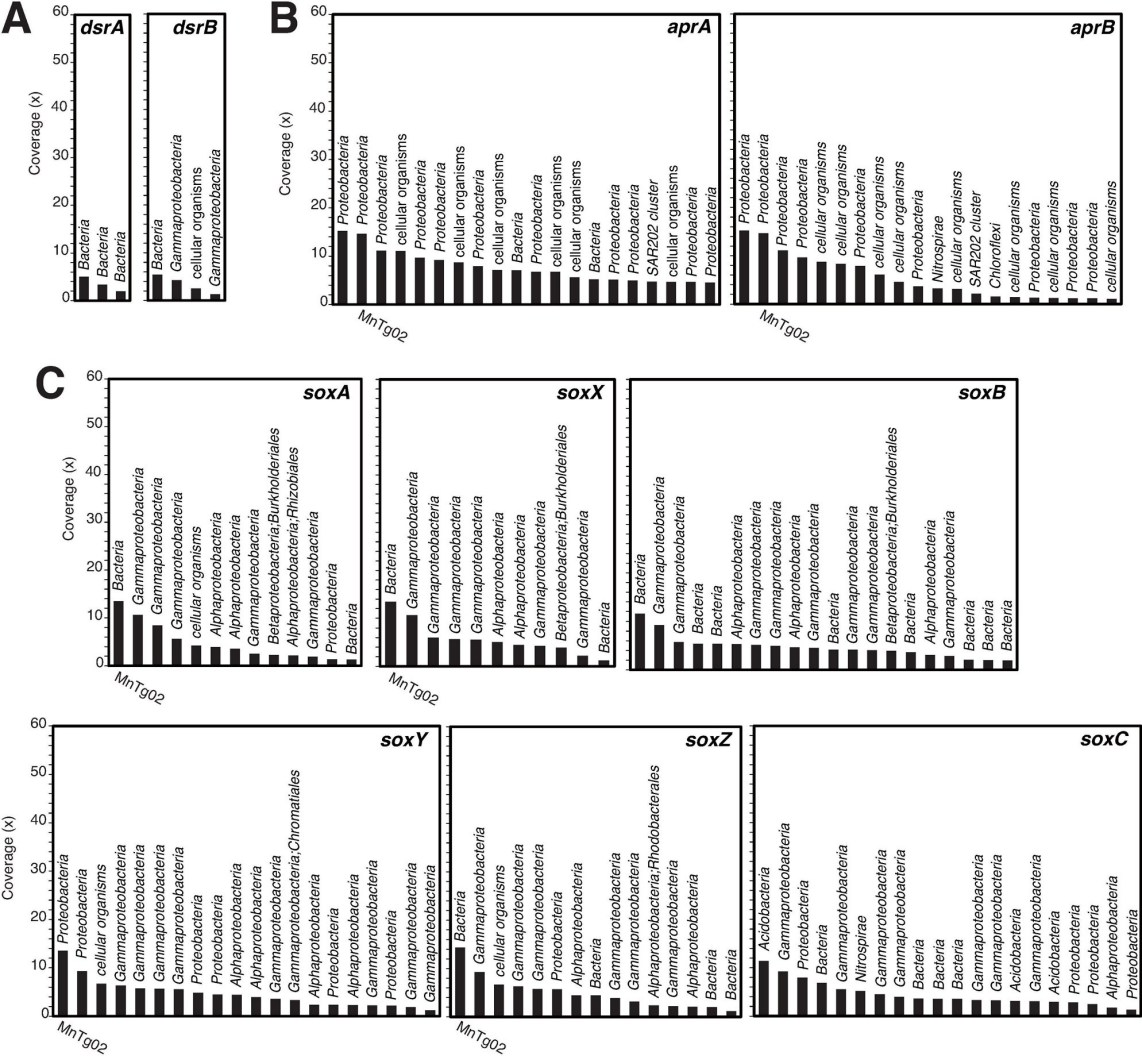
Read-coverage-based rank abundant plot of contigs containing CDSs potentially involved in sulfur oxidation. CDSs of (A) DsrAB, (B) AprAB, and (C) SoxABCXYZ. Taxonomic affiliations for the CDSs are indicated on the bars. The IDs of metagenome-assembled genomes (MAGs) are shown for the contigs containing CDSs identical to those of the MAGs.

Reduced sulfur species (such as hydrogen sulfide and thiosulfate) are another potential energy source for chemolithotrophs in the Fe-Mn crust. The reduced sulfur species can be supplied by microbial decomposition of sinking organic particulates containing sulfur. In the metagenome, we found CDSs for dissimilatory sulfite reductase (Dsr) [EC:1.8.99.5] and adenylyl-sulfate reductase (Apr) [EC:1.8.99.2], which are involved in sulfur oxidation or sulfate reduction (Fig 3A and 3B). Phylogenetic analyses showed that the detected CDSs for DsrA and AprA were clustered with those from sulfur-oxidizing bacteria (SOB) (S5 Fig), suggesting that these CDSs originated from SOB. We also found CDSs for the SOX system (SoxABCXYZ) (Fig 3C), which is involved in thiosulfate oxidation. The detection of these CDSs suggested the presence of SOB in the Fe-Mn crust. Read coverages of some of the CDSs were relatively high (>10×), indicating that some SOB were not rare members in the microbial community in the Fe-Mn crust.

**Fig 4:**
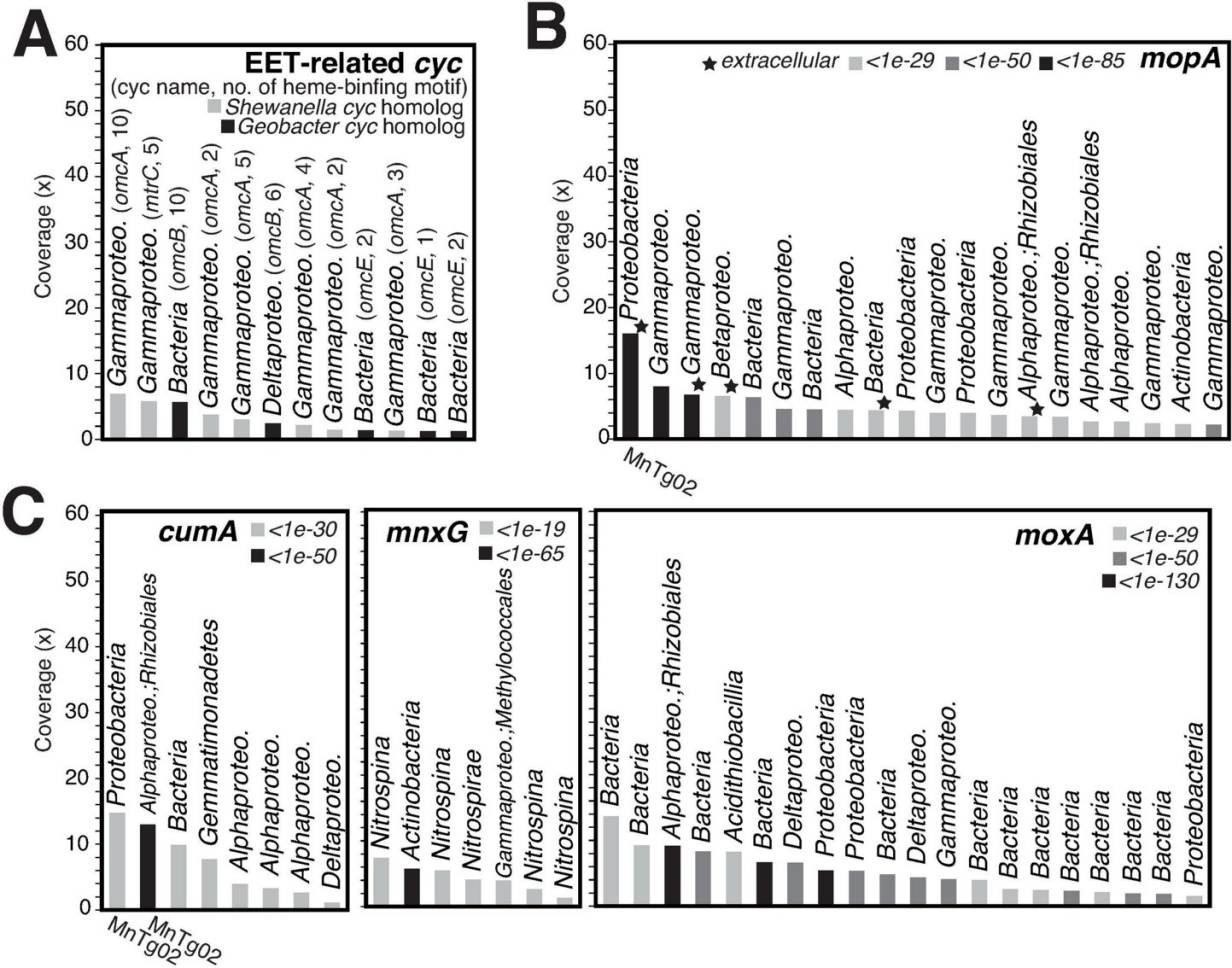
Read-coverage-based rank abundant plot of contigs containing CDSs potentially involved in Mn and Fe cycling. (A) CDSs for extracellular electron transfer (EET) related Cyc. The homologous gene name and the number of heme-binding motifs (CXXCH) are indicated in parentheses. CDSs for manganese oxidizing enzymes (B) MopA, and (C) CumA, MnxG, and MoxA. The threshold of BLAST e-values against the enzymes MopA of *Aurantimonas manganoxydans* SI85-9A1 (WP_009209951), CumA of *Pseudomonas putida* GB-1 (Accession no. AAD24211), MnxG of *Bacillus* sp. SG-1 (AAB06489), MoxA of *Pedomicrobium* sp. ACM 3067 (CAJ19378) are shown by the colors of the bars. The IDs of metagenome-assembled genomes (MAGs) are shown for the contigs containing CDSs identical to those of the MAGs.

Some microorganisms can reduce solid Fe(III) and Mn(IV) oxides or oxidize dissolved Fe^2+^ and Mn^2+^ in the crusts. For *Shewanella* spp. in *Gammaproteobacteria* and *Geobacter* spp. in *Deltaproteobacteria*, multiheme *c-*type cytochromes (Cyc) located in their outer membrane, such as MtrC and OmcA, are involved in the reduction of Fe(III) and Mn(IV) oxides via extracellular electron transfer [59–62]. The CDSs with one or multiple (up to ten) heme-binding motifs (CXXCH) showing high similarity to these outer membrane Cyc of *Shewanella* spp. and *Geobacter* spp. were found in the crust metagenome, although the read coverages were low (1× to 7×) (Fig 4A). OTUs belonging to *Shewanella* were detected in the above 16S rRNA gene amplicon analysis, although the abundance was also low (< 0.003% of the total reads from MnT55a) [13]. No OTUs related to *Geobacter* were detected in this sample. Outer membrane Cyc has been also suggested to be involved in Fe oxidation by Fe-oxidizing bacteria [63–65]. However, such CDSs were not found in the metagenome, which was consistent with the result that no OTUs related to neutrophilic Fe-oxidizing bacteria, such as *Mariprofundus* [66] and *Ferriphaselus* [67], were detected in the sample.

Regarding Mn oxidation, we found CDSs homologous to some Mn oxidases, such as MopA (Fig 4B), classified in a heme peroxidase family, and CumA, MnxG, and MoxA (Fig 4C) in a multicopper oxidase family [68, 69]. These Mn oxidases have been reported in *Aurantimonas* spp., *Pedomicrobium* spp., *Erythrobacter* spp. in *Alphaproteobacteria*, *Pseudomonas* spp. in *Gammaproteobacteria*, and *Bacillus* spp. in *Firmicutes*. In 16S rRNA gene amplicon analysis, the OTUs belonging to *Pseudomonas* and *Bacillus* were detected in this sample (0.09% and 0.08% of the total reads from MnT55a, respectively) [13]. Some MopA CDSs were estimated to be in the extracellular region, which is consistent with a previous report [70]. The BLASTp *e*-values for some Mn oxidase CDSs were relatively low (< 1e-130, in some cases), suggesting that the CDSs included Mn oxidases. Furthermore, some MopA CDSs were homologous to animal heme peroxidases found in *Roseobacter* sp. AzwK-3b, which is involved in Mn oxidation via superoxide production and H_2_O_2_ degradation [71]. In the production of Mn oxides (MnO_2_) via superoxide production, H_2_O_2_ degradation is required, as intermediate Mn(III) in Mn(II) oxidation can be reduced back to Mn(II) by H_2_O_2_ [72]. In the crust environments, H_2_O_2_ could be removed by MnO_2_ abiotically and by microorganisms possessing the H_2_O_2_ degrading enzyme catalase [EC 1.11.1.6], of which CDSs were found in the metagenome (S6 Fig), in addition to the animal heme peroxidase.

### Metabolic potential of metagenome-assembled genomes

**Fig 5:**
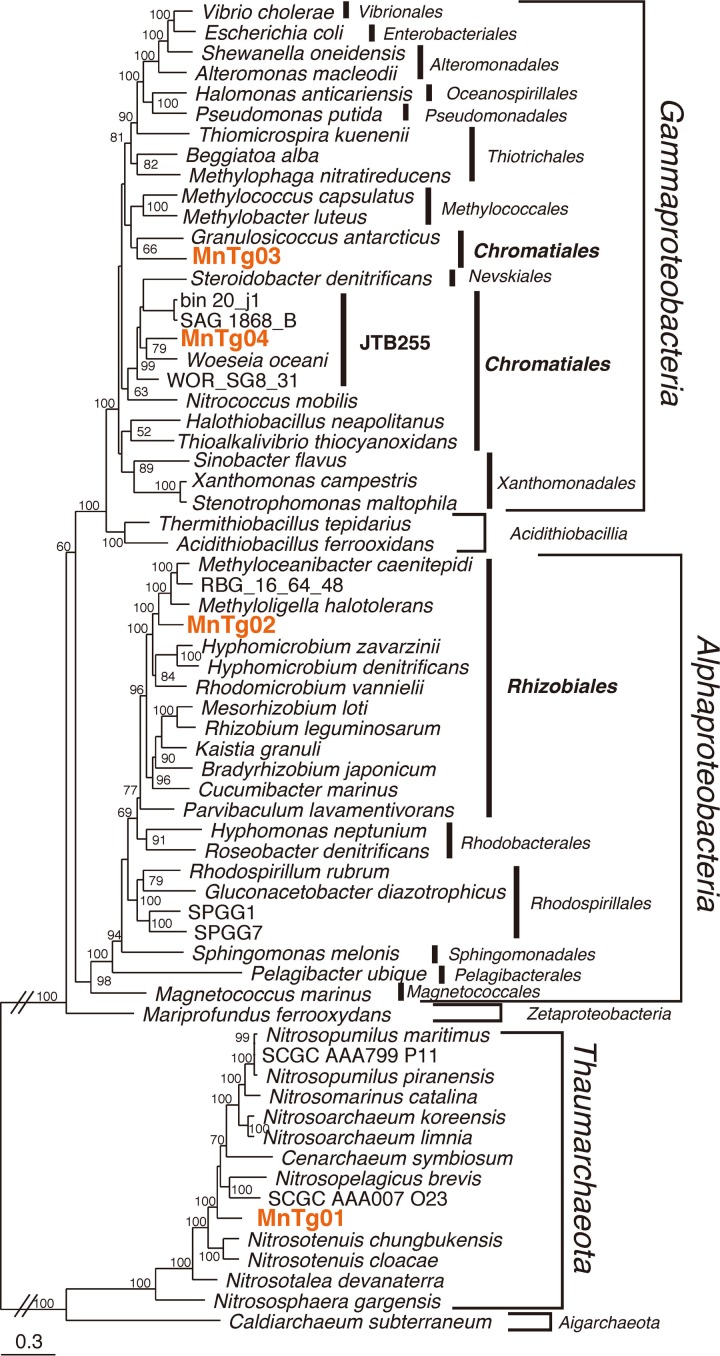
Phylogenomic tree for the MAGs. The maximum-likelihood tree of the MAGs was constructed using 43 conserved single-copy marker proteins. The IDs in bold and orange were reconstructed in the present study. Accession numbers for reference sequences are shown in S2 Table. The scale bar represents 0.3 amino acid substitutions per sequence position. Bootstrap values (> 50% of 1000 replicates) are indicated at nodes.

**Fig 6:**
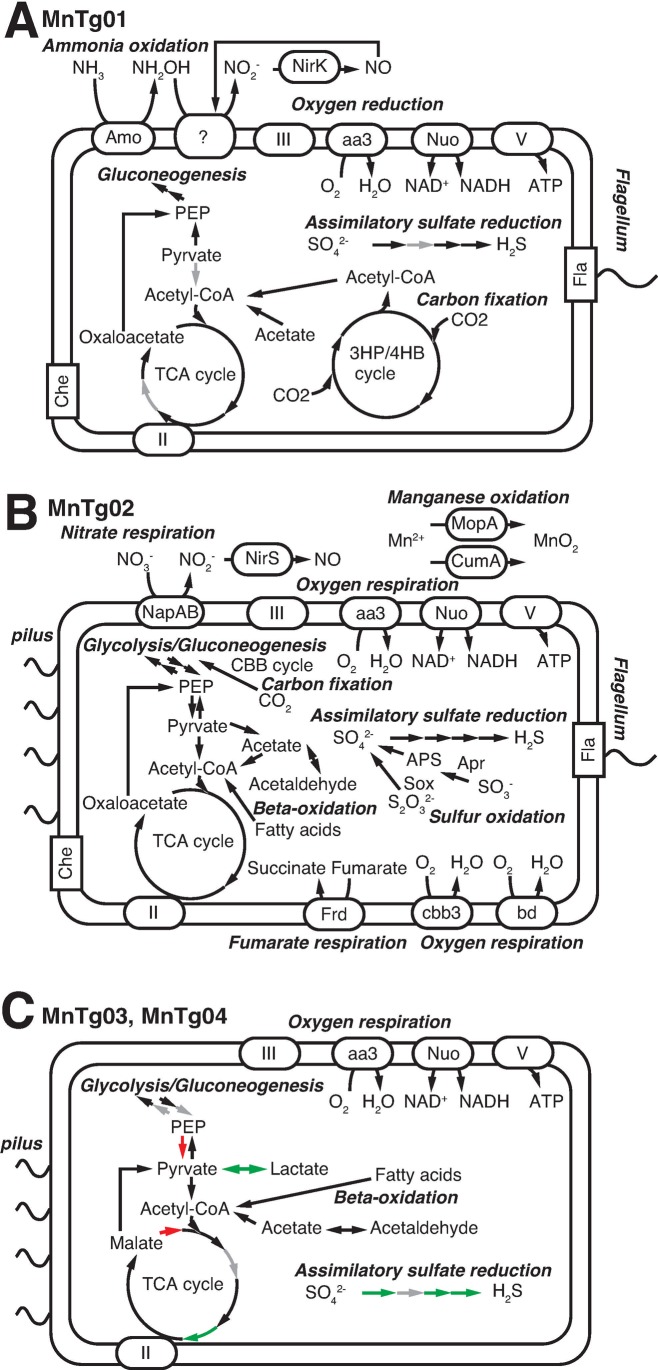
Key metabolic pathways of the four MAGs. (A) MnTg01, (B) MnTg02, (C) MnTg03 and MnTg04. (A and B) The presence and absence of each gene is indicated by black and gray arrows, respectively. (C) Black arrows indicate genes present in MnTg03 and MnTg04. White arrows indicate genes absent in MnTg03 and MnTg04. Green or red arrows indicate genes present only in MnTg03 or MnTg04, respectively. The locus tags and full names of the genes in the pathways are shown in S1 Table.

We obtained four metagenome-assembled genomes (MAGs), defined as MnTg01–04, with over 70% genome completeness and below 5% contamination (Table 1). All the MAGs were classified as medium-quality based on the standard minimum information about a metagenome-assembled genome (MIMAG) [73]. A phylogenetic tree of a concatenated amino acid sequence of CDSs for 43 single-copy marker proteins (Fig 5) showed that these MAGs were affiliated with *Thaumarchaeota* for MnTg01, *Alphaproteobacteria* for MnTg02, and *Gammaproteobacteria* for MnTg03 and MnTg04. The average read coverages for the CDSs for RpsC of the MAGs (Fig 1A) indicated that the MnTg01 thaumarchaeon was the most abundant member of the crust community. The MAGs of MnTg02 and MnTg04 were also within the top 10 members. Although no CDS for RpsC was found in MnTg03, one of the relatively abundant contigs containing gammaproteobacterial RpsC CDS (Fig 1A) was likely derived from the MnTg03 bacterium based on the read coverage of the MAG (Table 1) and phylogeny (Figs 5 and S1). The reconstructed metabolic pathways for the four MAGs are shown in Fig 6A–6D, and the CDSs involved in the pathways are listed in S1 Table.

**Table 1:** Summary of sequence information of the MAGs.

MAG ID		MnTg01	MnTg02	MnTg03	MnTg04
Taxonomy		Thaumarchaeota; MGI	Alphaproteobacteria; Rhizobiales	Gammaproteobacteria; Chromatiales	Gammaproteobacteria; Chromatiales; JTB255
Average coverage (x)		78.9	16.7	24.2	14.1
*Genomic feature*					
	Number of contigs	180	378	343	244
	MAG size (bp)	1,071,832	3,616,715	1,681,212	1,733,001
	Estimated genome size (Mbp)^a^	1.52	4.17	2.29	2.22
	G+C content	33.2%	54.6%	49.2%	58.0%
	Number of rRNA genes	16S x1	5S x1	N.D.	N.D.
	Number of tRNA genes	21	36	20	31
	Number of CDSs	1298	3468	1623	1752
	Longest contig (bp)	23610	42450	16445	31027
	N50 (bp)	6643	12880	5226	8580
	Completeness^b^	70.64%	86.78%	73.48%	78.13%
	Contamination^b^	1.46%	0%	4.47%	0.16%
*Metabolic potential*					
	Autotrophy	3HP/4HB cycle	CBB cycle	No	No
	Lithotrophy	Ammonia oxidation	Sulfur oxidation	No	No
	Heterotrophy	No	Yes	Yes	Yes
	Respiration	Aerobic	Aerobic, Anaerobic (Nitrate, Fumarate)	Aerobic	Aerobic

^a^Calculated based on genome completeness;

^b^Calculated by CheckM

The phylogenetic analysis (Figs 5 and S1) revealed that MnTg01 thaumarchaeon was located in the AOA clade which includes *Nitrosopumilus* spp. [74] and *Nitrosopelagicus* spp. [75]. Average amino acid identities (AAI) of MnTg01 to the cultivated AOA genomes were < 66.5%, suggesting that MnTg01 thaumarchaeon represented a novel genus based on a previously reported definition [76]. This was supported by the 93.7% sequence similarity of the 16S rRNA gene to the closest cultivated species, Candidatus *Nitrosopelagicus brevis* [75], which was lower than the 94.5% genus definition threshold [77]. The MnTg01 contained CDSs involved in ammonia oxidation (AmoABC and NirK), oxygen respiration (*aa*_*3*_-type Cox), and carbon fixation via the 3HP/4HB cycle (Fig 6A) as previously reported for thaumarchaeotic AOA [78, 79]. Many CDSs for the Embden–Meyerhof–Parnas (EMP) pathway and TCA cycle were found in MnTg01. CDSs involved in assimilatory sulfate reduction (Sat, CysH, and Sir) were also detected. No CDSs for GH families were detected. Notably, the MnTg01 contained CDSs for chemotaxis and archaeal flagellum (known as archaellum), as reported for some thaumarchaeotic AOA, such as *Nitrososphaera* spp. [79], *Nitrosotalea* spp. [80], and *Nitrosoarchaeum* spp. [81], suggesting that the MnTg01 thaumarchaeon had biofilm formation and surface adhesion capabilities [82].

The alphaproteobacterial MnTg02 was affiliated with the order *Rhizobiales* (Fig 5). This MAG was related to species in the genera *Methyloligella*, *Hyphomicrobium*, and *Rhodomicrobium* and to MAG RBG_16_64_48 recovered from terrestrial sediments [83] (AAI, < 56.3%), but clearly not to alphaproteobacterial MAGs (SPGG1 and SPGG7) recovered from pelagic deep-sea sediments [29]. Considering that an AAI threshold of 45–65% has been used for a family level definition [76], the alphaproteobacterial MnTg02 should be classified as a novel family. The MnTg02 contained CDSs for oxygen respiration (*aa*_*3*_-Cox, *cbb*_*3*_-Cox, and *bd*-type quinol oxidase), nitrate respiration (NapAB and NirS), fumarate respiration (Frd), thiosulfate/sulfite oxidation (SoxAXYZ, AprAB, and Sat), carbon fixation via the CBB cycle, sugar decomposition (GH families 3, 15, 23, 103 and 130), fatty acid decomposition (beta-oxidation pathway), and assimilatory sulfate reduction (CysNCDHI), in addition to the EMP pathway and TCA cycle (Fig 6B). As described above, CDSs for manganese oxidation (MopA and CumA) were also detected. The presence of CDSs for chemotaxis, pilus, and flagellum suggested biofilm formation and surface adhesion capabilities. In the biofilm, anoxic microenvironments could be created, where the MnTg02 alphaproteobacterium could grow by anaerobic respiration using nitrate and fumarate. In addition to the organic carbon in the sinking POC, reduced sulfur species, which could be produced by decomposition of sulfur-containing organic compounds (such as cysteine and methionine) contained in the POC, might be also used as an energy source for the MnTg02 alphaproteobacterium.

The two gammaproteobacterial members derived from the MAGs (MnTg03 and MnTg4) were affiliated with the order *Chromatiales* of the *Gammaproteobacteria* (Figs 5 and S1) and differed from each other at least at the family level (AAI, 45.4%). MnTg03 was distantly related to cultivated species of *Chromatiales* such as *Granulosicoccus*, *Thioalkalivibrio*, and *Halothiobacillus* with relatively low AAI values (< 47.2%). MnTg04 was affiliated with the clade JTB255/*Woeseiaceae*, whose members have been commonly detected in deep-sea sediments [84, 85]. MnTg04 was related to a chemoheterotrophic bacterium *Woeseia oceani* [86] and some MAGs/SAGs recovered from marine sediments [53, 85] (AAI, < 54.4%). Previous metagenomic studies and radio-isotope incubation experiments [85, 87] have suggested that the JTB255/*Woeseiaceae* contain autotrophs. The two gammaproteobacterial MAGs contained CDSs for the EMP pathway, TCA cycle, beta-oxidation pathway for lipid decomposition, GH families (3, 15, 23, 103 and 130) for sugar decomposition, and aerobic respiration, but none for chemolithoautotrophic metabolism or anaerobic respiration (Fig 6C and 6D). Therefore, they may be aerobic chemoheterotrophs and/or facultative anaerobic fermenting bacteria.

## Discussion

**Fig 7:**
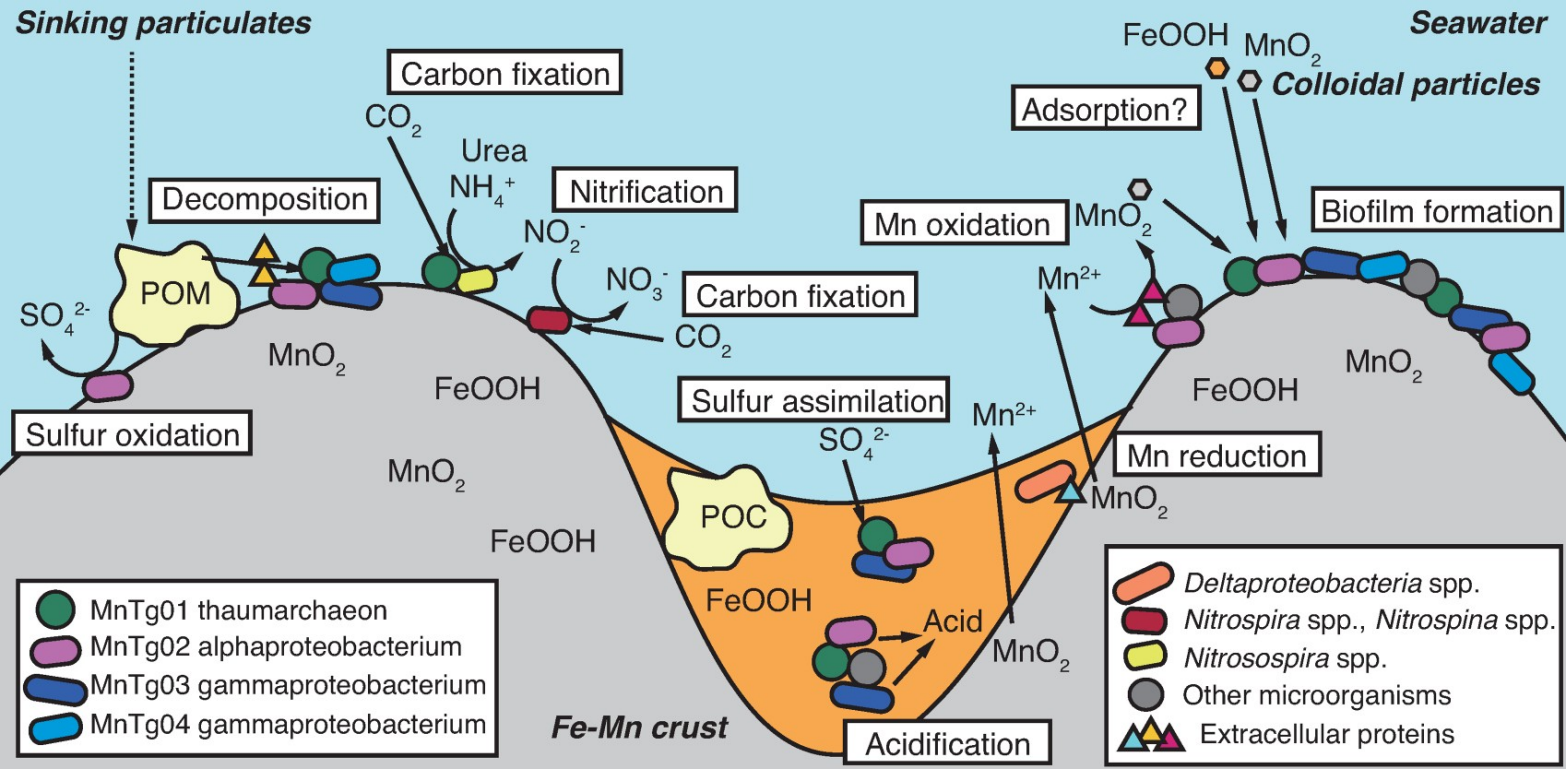
Proposed model of the microbial contribution to the biogeochemical cycling of C, N, S, Fe, and Mn and the formation of the Fe-Mn crust. Illustration of a side-view of the crust at micrometer-scale is shown. Microorganisms with nano-sized fibers form biofilms in the crusts [13]. The biofilm can adsorb Fe and Mn oxide particles in seawater. Extracellular proteins produced by the microorganisms contribute to decomposition of particulate organic matter (POM) and manganese oxidation/reduction. Nitrifers play a role as primary producers in the crust ecosystem. Acidification by fermenters and ammonia oxidizers cause dissolution of Mn and Fe oxides. See main text for details.

Our metagenomics data provided novel insights into the microbial ecology of the Fe-Mn crust surface, and to the microbial contribution to the formation of the Fe-Mn crust. Previous studies have shown the microbial community structures on the Fe-Mn crust surface using single-gene analysis (16S rRNA and *amoA* genes) [11, 13, 14]; however, their metabolic potentials were largely unknown. In the present study, we propose a model of the biogeochemical cycling of C, N, S, Fe and Mn on the crust surface (Fig 7) based on the metagenomic results, in addition to the previous microscopic results [13].

### Microbial ecosystem in the abyssal Fe-Mn crust

As an energy source, ammonia dissolved in seawater (generally several-tens nM or lower [88]) likely sustains the microbial ecosystem on the crust surface, as previously suggested [11, 13, 14]. It has been reported that ammonia is an important energy source in the deep ocean [89]. Based on the genes detected in the crust metagenome, nitrification (i.e., ammonia oxidation and nitrite oxidation) and carbon fixation are likely conducted by members of the MGI of *Thaumarchaeota*, *Nitrosomonadales* of *Betaproteobacteria*, *Nitrospirae*, and *Nitrospinae*. Furthermore, we obtained the MAG MnTg01 of an MGI member, which was the most abundant in the crust (Fig 1A). The genomic analysis of the MnTg01 confirmed the potential of ammonia oxidation by Amo and carbon fixation via the 3HP/4HB pathway (Fig 6A). Considering the high abundance of the MnTg01 in the microbial community and its chemolithoautotrophy potential, the MnTg01 thaumarchaeon likely plays a significant role as a primary producer in the microbial ecosystem of the Fe-Mn crust.

The gene survey in the metagenome suggests that urea is also used as an energy source by the Fe-Mn crust microorganisms. Previous isotope labeling studies have shown that urea is an important energy source for thaumarchaeotic AOA and betaproteobacterial AOB in pelagic oceans [90–92]. Previous physiological characterization has confirmed that urea is used for some of the AOA and AOB cultivated isolates as an energy and carbon source [93, 94]. Based on the read coverage and taxonomic affiliation of CDSs for Ure and RpsC (Figs 1A and 2A), several abundant members of *Thaumarchaeota* on the crust surface may use urea as an energy and carbon source. However, the most abundant thaumarchaeotic member (i.e., the MnTg01 archaeon; read coverage, >45×) may not use urea, because the CDS for Ure was not found in its MAG. This is supported by the fact that no CDSs for Ure in the metagenome showed high read coverage. Thus, urea may support the microbial ecosystem as a secondary energy source.

Although cyanate, a decomposition product of urea, is another potential energy source in oceans [92, 95], it may not be used as an energy source for the crust microorganisms. Several tens nM of cyanate have been detected in seawater [96]. Indeed, a thaumarchaeotic AOA isolate, *Candidatus* ‘Nitrososphaera gargensis’, of which the genome encodes Cyn and Amo, can grow with cyanate, which is converted into ammonia by Cyn, as the sole energy source [95]. However, we only found two CDSs for Cyn in the metagenome, and those CDSs were not affiliated with AOA or AOB. The gammaproteobacterial and alphaproteobacterial species possessing the CDSs for Cyn (Fig 2A) might use cyanate as a nitrogen source.

The Fe-Mn crust likely harbors heterotrophic microorganisms possessing GHs and peptidases, which likely use organic carbon derived from POC as their energy and carbon source. The MnTg03 and MnTg04 gammaproteobacteria likely contribute to the POC decomposition. Accordingly, the POC decomposition by heterotrophs can produce reduced sulfur compounds (such as sulfide and thiosulfate). The MnTg02 alphaproteobacterium and other bacteria, which possess the CDSs (such as Apr and Sox) involved in oxidation of reduced sulfur compounds, can chemolithotrophically use these sulfur compounds as energy sources. In addition, abiotic POC decomposition by Mn oxides into more labile carbon compounds such as pyruvate [58], which are easily used by heterotrophs, could occur in the Fe-Mn crust. Although there are several tens μM of dissolved organic carbon (DOC) in deep seawater, the majority of the DOC is refractory [97] and is not easily used by microorganisms [98]. However, if Mn oxides in the Fe-Mn crust also abiotically decompose such refractory DOC into labile organic compounds [58], the heterotrophic microorganisms can use them as energy and carbon sources.

Anaerobes may also be present in the Fe-Mn crust. Our previous microscopic observation indicated that microorganisms accumulated in valleys on the bumpy surface of the Fe-Mn crust [13]. Oxygen consumption by aerobes may create anoxic microenvironments in the valleys where POC are trapped. In such anoxic microenvironments, anaerobic respiration bacteria using nitrate, fumarate, Fe and Mn oxides as electron acceptors, and fermenting bacteria producing organic acids (such as lactic and acetic acids) can grow. This was supported by the detection of the CDSs involved in these anaerobic reactions in the metagenome.

Overall, the microbial community in the oligotrophic Fe-Mn crust is likely sustained by dissolved nitrogen compounds (i.e., ammonia and urea) and sinking particles containing organic carbon and sulfur as energy sources, which are suppled from the photosynthetic ecosystem at the ocean surface. Considering the wide distribution of the Fe-Mn crusts in oceans [2], the abundance and ubiquitousness of microorganisms in the crusts [11, 13], and their potential metabolic functions reported in this study, microbial communities in Fe-Mn crusts potentially play an important role in scavenging organic compounds for the whole ocean ecosystem.

### Biological contribution to geochemical cycling of Fe and Mn

The microbial metabolisms described above potentially contribute to geochemical cycling of Fe and Mn. For instance, ammonia oxidation by AOA and AOB could decrease the pH by the following reaction: NH_4_^+^ + 1.5 O_2_ → NO_2_^-^ + H_2_O + 2 H^+^. Organic acid production by fermenting bacteria could also decrease the pH. Because Mn^2+^ is more thermodynamically stable than Mn oxides (as MnO_2_) at lower pH (<7) even under aerobic conditions, a decreased pH could cause dissolution of Mn oxides in the Fe-Mn crusts. The putative Mn reducers possessing extracellular Cyc could also be involved in the Mn reduction and dissolution. These microbial Mn dissolution processes may explain the Mn depletion in the valleys on the Fe-Mn crust surface, which have been shown by a previous microscopic observation [13]. Fe oxides can be also reduced and dissolved by putative Fe reducers possessing extracellular Cyc, but the dissolved Fe^2+^ can be rapidly re-oxidized to Fe oxides abiotically under the aerobic and weak alkaline conditions of seawater.

The dissolved Mn^2+^ can be oxidized in the Fe-Mn crust by putative Mn oxidizers possessing Mn oxidases, such as the MnTg02 alphaproteobacterium. In addition, diverse microorganisms with H_2_O_2_-degrading capability could also be involved in Mn oxidation, as reported previously [71, 99]. Some microorganisms including members in the MGI of *Thaumarchaeota* and *Rhizobiales* of *Alphaproteobacteria* may form biofilms on the Fe-Mn crust. In fact, microbial cells with fibrous organic materials covering the crust surface have been observed by scanning electron microscopy [13]. Such biofilms can adsorb Mn and Fe oxide colloidal particles [100, 101].

The above microbial activities could contribute to both the acceleration and suppression of the growth of the Fe-Mn crust. The extremely slow growth rate of Fe-Mn crusts (1–10 mm/Myr) is potentially controlled by the balance of the acceleration and suppression of oxidation/reduction and dissolution/precipitation of Mn and Fe by microbial activities, although further experimental evaluation is needed.

## Conclusions

The first metagenomic analysis of a deep-sea Fe-Mn crust indicated the potential of biogeochemical cycling of C, N, S, Fe, and Mn, driven by as-yet-uncultivated microorganisms. Our results led us to propose a model of the biogeochemical cycling in deep-sea Fe-Mn crusts, which implies that the growth rate of the Fe-Mn crusts is constrained by microbial activities. However, it remains unclear how active the metabolisms of these microorganisms are *in situ* environments, and how significant their activities are in oceanic biogeochemical cycling. Further studies, such as metatranscriptomics/metaproteomics and *in situ* tracer experiments, will be necessary to address the above questions.

## Supporting information

S1 Fig Phylogenetic tree of RpsC detected in the metagenome. The IDs in orange and green were detected in the metagenome and the MAG MnTg01, respectively. Numbering (#1 to #20) of the top 20 of the higher read coverages are shown. The RpsC sequences from #10 and #12 in *Alphaproteobacteria* were removed from the tree construction because of their short sequences. The scale bar represents 0.1 amino acid substitutions per sequence position. Bootstrap values (> 50% of 1000 replicates) are indicated at nodes.
(PDF)

S2 Fig Phylogenetic tree of NxrB/NarH. The IDs in orange were detected in the metagenome. The clades of nitrite oxidizers are indicated. The scale bar represents 0.3 amino acid substitutions per sequence position. Bootstrap values (> 50% of 1000 replicates) are indicated at nodes.
(PDF)

S3 Fig Rank abundant plot of CDSs for GH families detected in the metagenome. (PDF)

S4 Fig Read-coverage-based rank abundant plot of contigs containing CDSs for formaldehyde dehydrogenase (Fdh) and lactate dehydrogenase (Ldh). Taxonomic affiliations for the genes are indicated on the bars.
(PDF)

S5 Fig Phylogenetic tree of DsrA and AprA. The IDs in orange were detected in the metagenome. The clades of sulfur oxidizers and sulfate reducers are indicated. The scale bar represents 0.1 amino acid substitutions per sequence position. Bootstrap values (> 50% of 1000 replicates) are indicated at nodes.
(PDF)

S6 Fig Read-coverage-based rank abundant plot of contigs containing CDSs for catalases (KatE and KatG). Taxonomic affiliations for the genes are indicated on the bars.
(PDF)

S1 Table List of CDSs encoded in the MAGs. (PDF)

S2 Table List of accession numbers used in Fig 1. (PDF)
