# Supplementary figures and images for: Microbial metabolisms in an abyssal ferromanganese crust from the Takuyo-Daigo Seamount as revealed by metagenomics
Source: PLoS One. 2019 Nov 8;14(11):e0224888. doi: 10.1371/journal.pone.0224888 (PMC6839870; doi:10.1371/journal.pone.0224888)

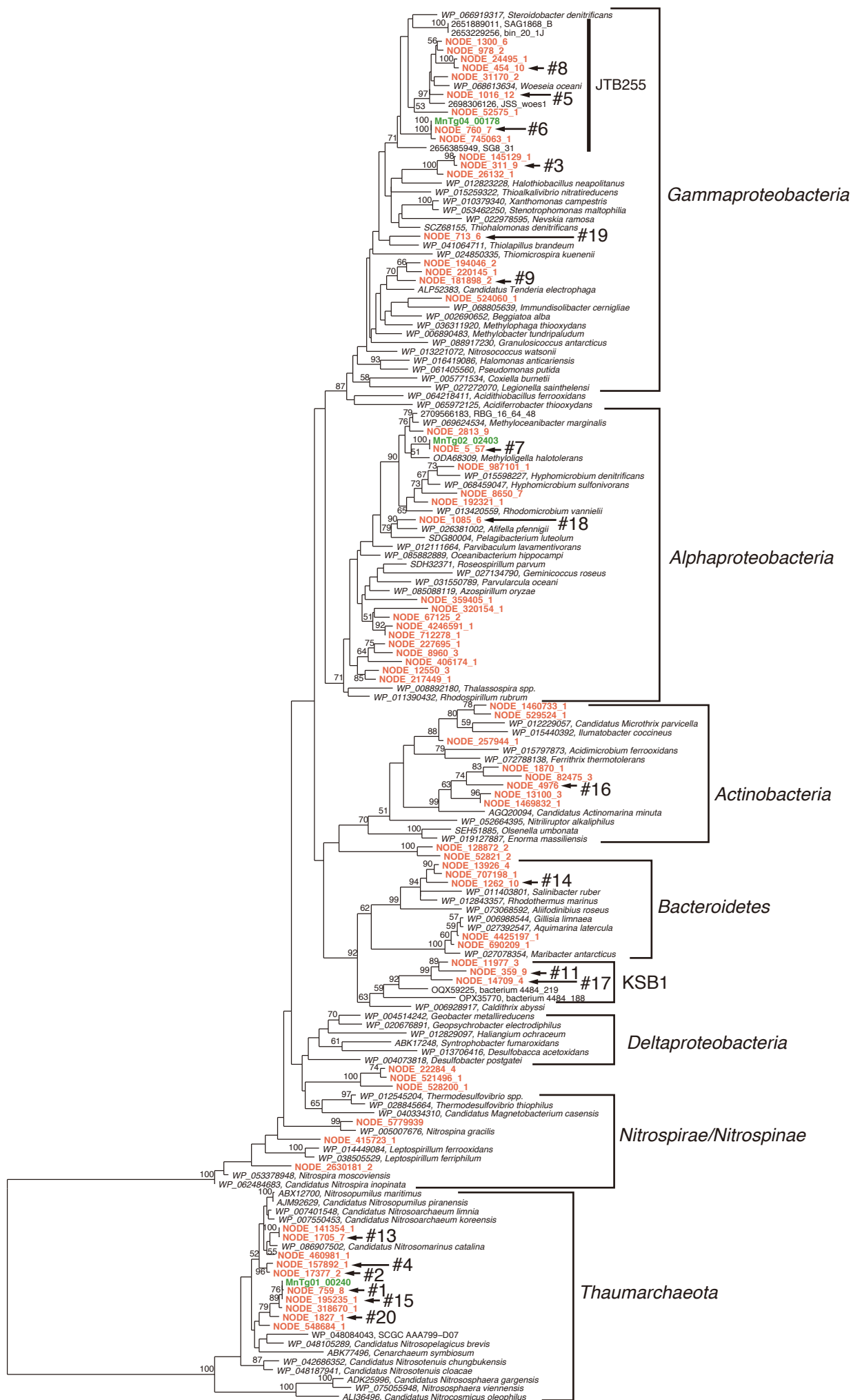

Supplement: S1 Fig — The IDs in orange and green were detected in the metagenome and the MAG MnTg01, respectively. Numbering (#1 to #20) of the top 20 of the higher read coverages are shown. The RpsC sequences from #10 and #12 in Alphaproteobacteria were removed from the tree construction because of their short sequences. The scale bar represents 0.1 amino acid substitutions per sequence position. Bootstrap values (> 50% of 1000 replicates) are indicated at nodes. (PDF) [file pone.0224888.s001.pdf]

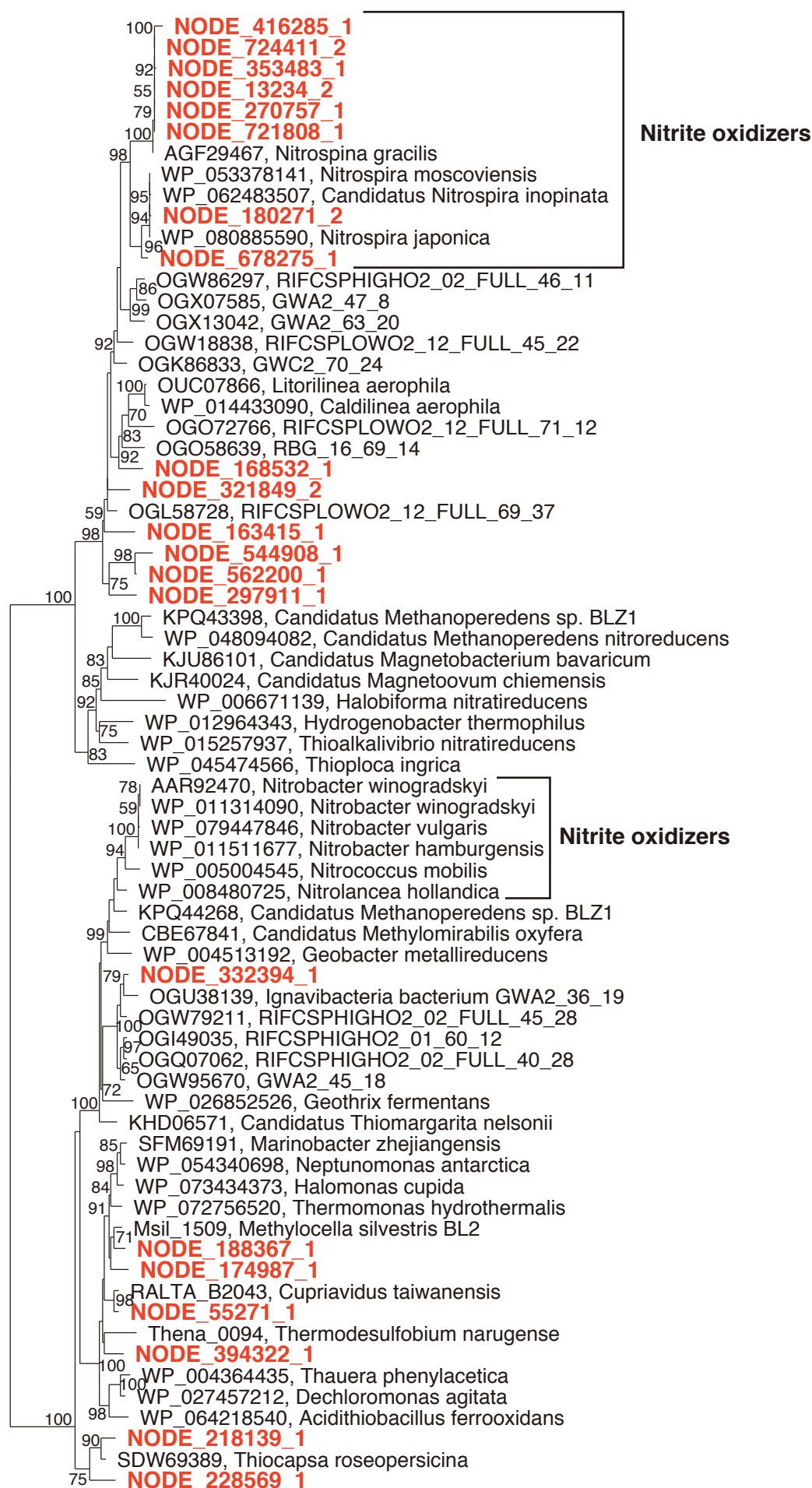

S2 Fig

Supplement: S2 Fig — The IDs in orange were detected in the metagenome. The clades of nitrite oxidizers are indicated. The scale bar represents 0.3 amino acid substitutions per sequence position. Bootstrap values (> 50% of 1000 replicates) are indicated at nodes. (PDF) [file pone.0224888.s002.pdf]

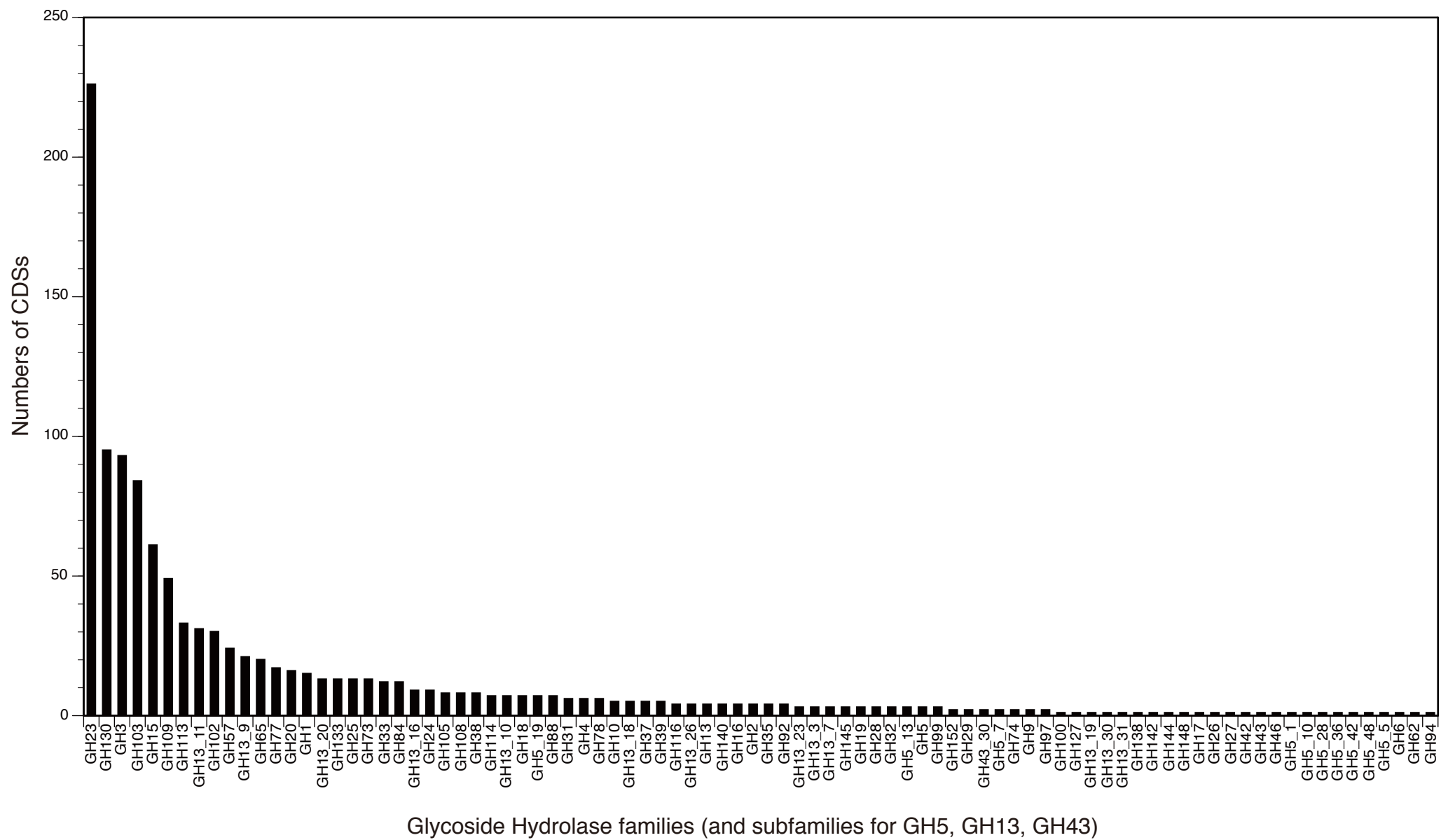

S3 Fig

Supplement: S3 Fig — (PDF) [file pone.0224888.s003.pdf]

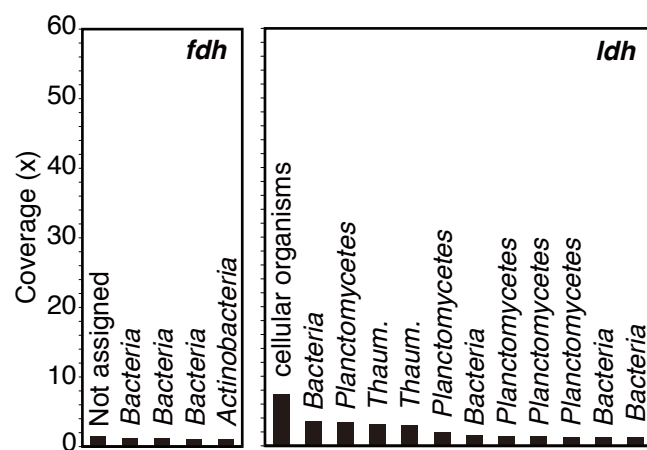

**S4 Fig**

Supplement: S4 Fig — Taxonomic affiliations for the genes are indicated on the bars. (PDF) [file pone.0224888.s004.pdf]

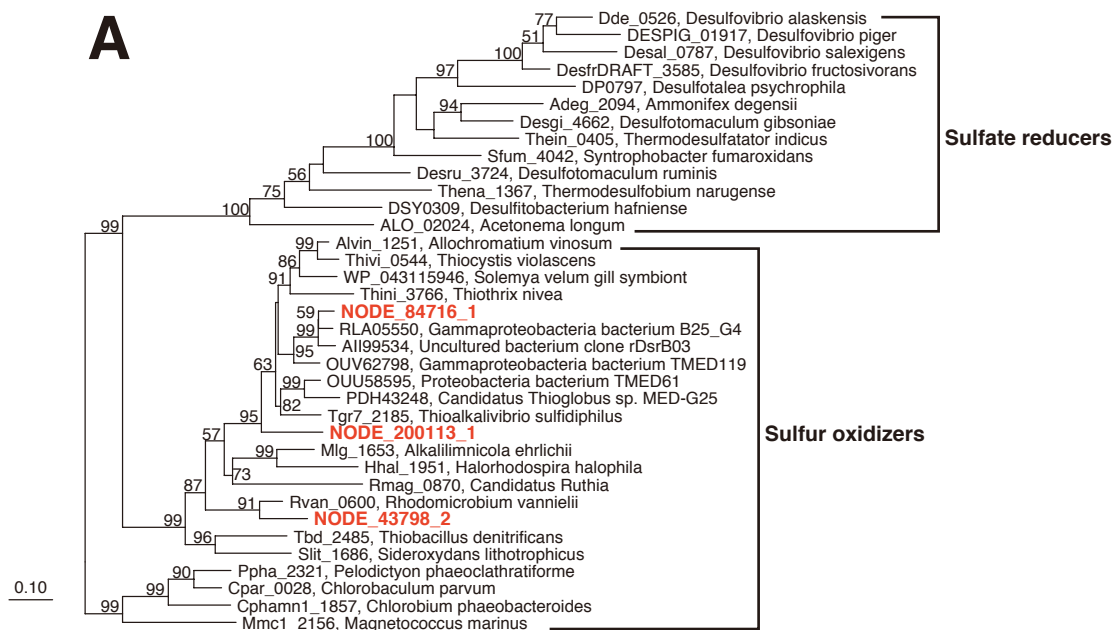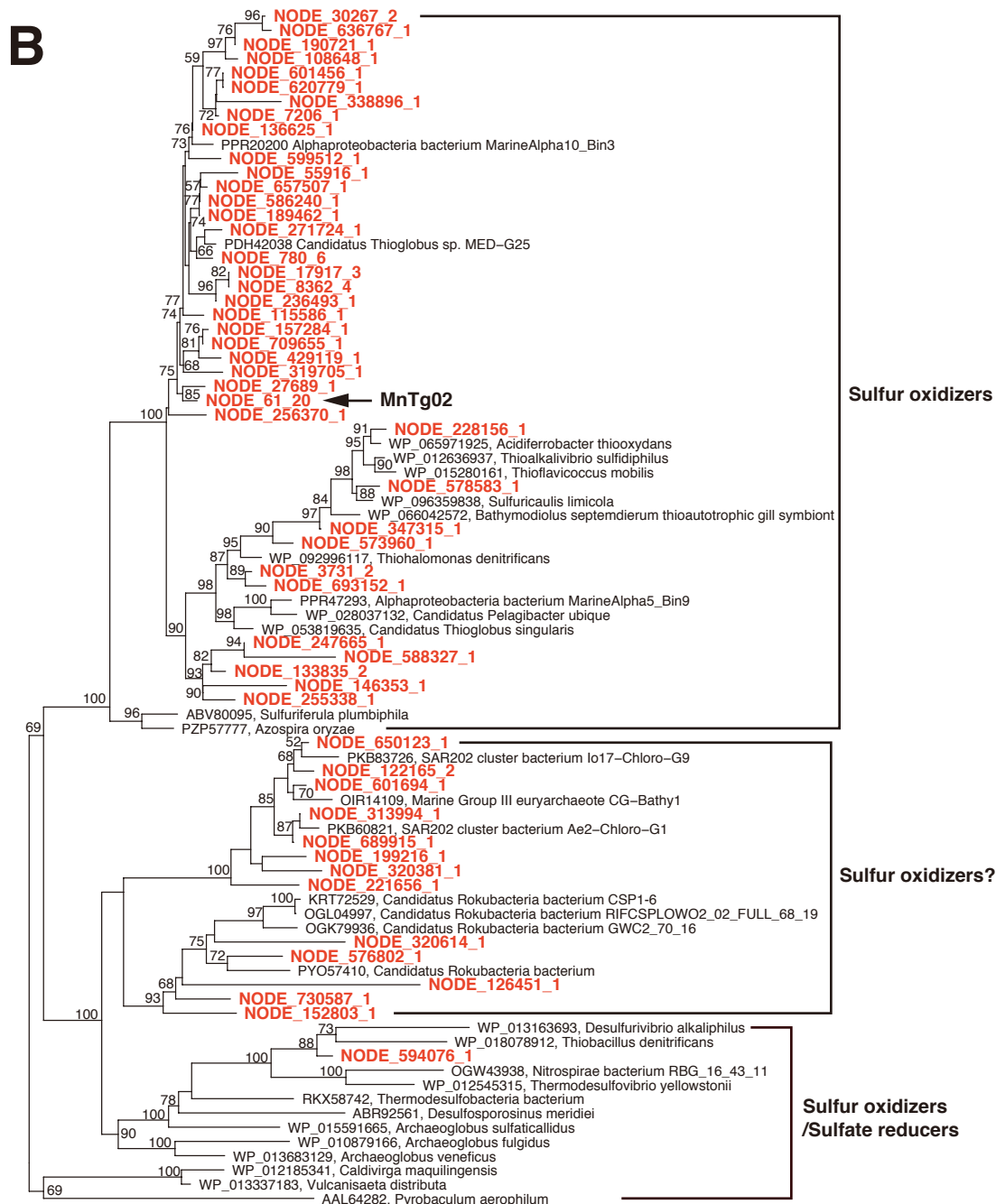

**S5 Fig**

Supplement: S5 Fig — The IDs in orange were detected in the metagenome. The clades of sulfur oxidizers and sulfate reducers are indicated. The scale bar represents 0.1 amino acid substitutions per sequence position. Bootstrap values (> 50% of 1000 replicates) are indicated at nodes. (PDF) [file pone.0224888.s005.pdf]

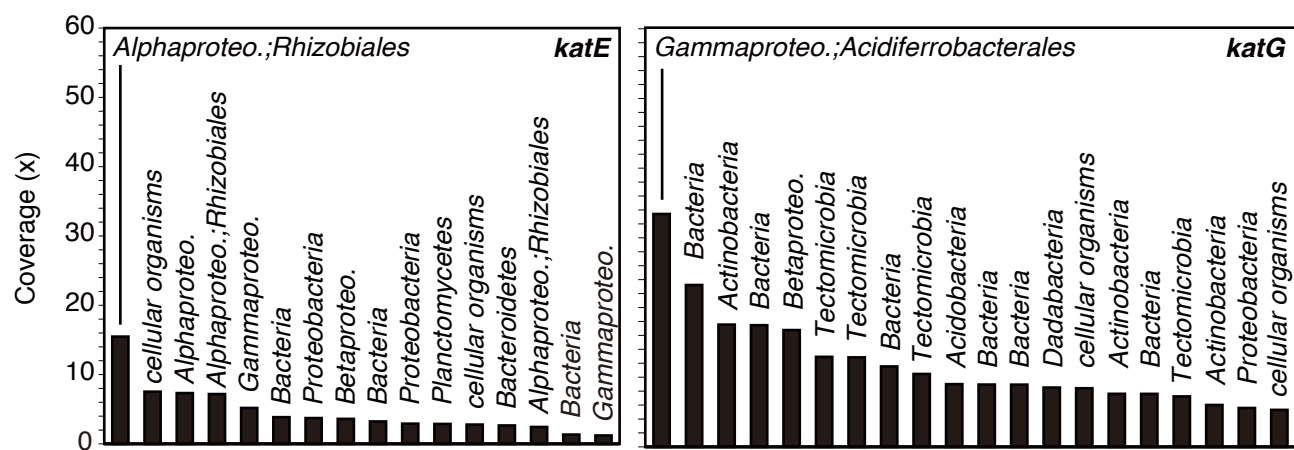

**S6 Fig**

Supplement: S6 Fig — Taxonomic affiliations for the genes are indicated on the bars. (PDF) [file pone.0224888.s006.pdf]
