# Supplementary material for: Microbial metabolisms in an abyssal ferromanganese crust from the Takuyo-Daigo Seamount as revealed by metagenomics
Source: PLoS One. 2019 Nov 8;14(11):e0224888. doi: 10.1371/journal.pone.0224888 (PMC6839870; doi:10.1371/journal.pone.0224888)
Supplement: S1 Table — (PDF) [file pone.0224888.s007.pdf]

S1 Table. List of CDSs encoded in the MAGs

		Thaumarchaeota	Alphaproteobacteria	Gammaproteobacteria	
KEGG#	Gene	MnTg01	MnTg02	MnTg03	MnTg04
**Manganese metabolism**					
**Manganese oxidation**					
-	*mopA*		MnTg02_02537		
-	*cumA*		MnTg02_00793, MnTg02_01158		
**Sulfur metabolism**					
**Assimilatory sulfate reduction, sulfate => H2S**					
sulfate adenylyltransferase [EC:2.7.7.4]					
K00958	*sat*	MnTg01_00681	MnTg02_01343	MnTg03_01267	
K00955	*cysNC*		MnTg02_02119		
K00957	*cysD*		MnTg02_02120		
phosphoadenosine phosphosulfate reductase [EC:1.8.4.8, EC 1.8.4.10]					
K00390	*cysH*	MnTg01_00682	MnTg02_00206	MnTg03_01042	
sulfite reductase [EC:1.8.1.2, EC:1.8.7.1]					
K00381	*cysI*		MnTg02_02440	MnTg03_01039	
K00392	*sir*	MnTg01_00991			
**Dissimilatory sulfate reduction, sulfate => H2S**					
sulfate adenylyltransferase [EC:2.7.7.4]					
K00958	*sat*	MnTg01_00681	MnTg02_01343	MnTg03_01267	
adenylylsulfate reductase [EC:1.8.99.2]					
K00394	*aprA*		MnTg02_01346		
K00395	*aprB*		MnTg02_01345		
**Thiosulfate oxidation by SOX complex, thiosulfate => sulfate**					
K17222	*soxA*		MnTg02_01156		
K17223	*soxX*		MnTg02_00610		
K17226	*soxY*		MnTg02_00654, MnTg02_01087		
K17227	*soxZ*		MnTg02_00655		
**Nitrogen metabolism**					
**Dissimilatory nitrate reduction, nitrate => ammonia**					
periplasmic nitrate reductase [EC:1.7.99.4]					
K02567	*napA*		MnTg02_02280		
K02568	*napB*				
assimilatory nitrate reductase [EC:1.7.99.-]					
K00372	*nasA*		MnTg02_01838		
nitrite reductase [EC:1.7.2.1]					
K00368	*nirK*	MnTg01_00454, MnTg01_00455			
K15864	*nirS*		MnTg02_01127		
**Ammonia oxidation, ammonia => nitrite**					
periplasmic nitrate reductase [EC:1.7.99.4]					
K10944	*amoA*	MnTg01_00174			
K10945	*amoB*	MnTg01_00171			
K10946	*amoC*	MnTg01_00172			
**Carbon metabolism**					
**Calvin–Benson–Bassham cycle**					
phosphoribulokinase [EC:2.7.1.19]					
K00855	*prk*		MnTg02_01679		
ribulose-bisphosphate carboxylase [EC:4.1.1.39]					
K01601	*rbcL*		MnTg02_01676		
K01602	*rbcS*		MnTg02_01675		
**3-hydroxypropionate/4-hydroxybutyrate cycle**					
acetyl-CoA carboxylase [EC 6.4.1.2]					
K01961	*accC*	MnTg01_00467	MnTg02_02072, MnTg02_02979	MnTg03_01361	MnTg04_01387

K01962	*accA*	MnTg01_00466	MnTg02_02067		MnTg04_00040
K01963	*accB*	MnTg01_00468, MnTg01_00573			
**Formate oxidation, formate => CO2**					
formate dehydrogenase [EC:1.2.1.2]					
K00122	*fdh*		MnTg02_00185		
K00123	*fdoG*		MnTg02_00870, MnTg02_01821	MnTg03_00735	
K00124	*fdoH*		MnTg02_01820	MnTg03_00734	
K00127	*fdoI*		MnTg02_01818	MnTg03_00733	
**Phosphate acetyltransferase-acetate kinase pathway, acetyl-CoA => acetate**					
phosphate acetyltransferase [EC:2.3.1.8]					
K00625	*pta*		MnTg02_03101		
**Acetate assimilation, acetate => acetyl-CoA**					
acetyl-CoA synthetase [EC:6.2.1.1]					
K01895	*acs*	MnTg01_00363	MnTg02_03373	MnTg03_00572, MnTg03_01305	MnTg04_00051
**Glycolysis (Embden-Meyerhof pathway), glucose => pyruvate**					
glucokinase [EC:2.7.1.2]/polyphosphate glucokinase [EC:2.7.1.63]					
K00845	*glk*		MnTg02_02779		
glucose-6-phosphate isomerase [EC:5.3.1.9, EC:2.2.1.2, EC:5.3.1.8]					
K01810	*pgi*		MnTg02_00715		
K15916	*pgi-pmi*	MnTg01_00133			
fructose-bisphosphate aldolase [EC:4.1.2.13, EC:4.1.2.13, EC:2.2.1.11]					
K01623	*aldo*		MnTg02_03036		MnTg04_01321
K01624	*fbaA*		MnTg02_01677		
triosephosphate isomerase (TIM) [EC:5.3.1.1]					
K01803	*tpi*		MnTg02_03212		MnTg04_00560
glyceraldehyde 3-phosphate dehydrogenase [EC:1.2.1.12]					
K00134	*gapA*		MnTg02_03035		MnTg04_00392
K00150	*gap2*	MnTg01_00815			
phosphoglycerate kinase [EC:2.7.2.3]					
K00927	*pgk*	MnTg01_00759	MnTg02_00608		MnTg04_00393
2,3-bisphosphoglycerate-dependent phosphoglycerate mutase [EC:5.4.2.11, EC:5.4.2.12]					
K01834	*gpmA*				MnTg04_00481
K15633	*gpmI*			MnTg03_00143	
K15634	*gpmB*	MnTg01_00143	MnTg02_01879	MnTg03_00687, MnTg03_00702	
K15635	*apgM*	MnTg01_00630			
enolase [EC:4.2.1.11]					
K01689	*eno*	MnTg01_00094	MnTg02_02925		
pyruvate kinase [EC:2.7.1.40]					
K00873	*pyk*		MnTg02_01825		MnTg04_00394
**Gluconeogenesis, oxaloacetate => fructose-6P**					
phosphoenolpyruvate carboxykinase [EC:4.1.1.32, EC:4.1.1.49]					
K01596	*pck*				MnTg04_01186
K01610	*pck*	MnTg01_01228	MnTg02_03253		
fructose-1,6-bisphosphatase [EC:3.1.3.11]					
K03841	*fbp*		MnTg02_01680		
K11532	*glpX*		MnTg02_01409		
fructose 1,6-bisphosphate aldolase/phosphatase [EC:4.1.2.13 3.1.3.11]					
K01622	*fbap*	MnTg01_00119			
**Pyruvate oxidation, pyruvate => acetyl-CoA**					
pyruvate dehydrogenase [EC:1.2.4.1]					
K00163	*aceE*		MnTg02_00446, MnTg02_03100	MnTg03_01038	MnTg04_01410
K00161	*pdhA*		MnTg02_02923		MnTg04_00878
K00162	*pdhB*		MnTg02_02922		MnTg04_00879

K00627	*pdhC*		MnTg02_02920, MnTg02_03099	MnTg03_01037	MnTg04_01409
K00382	*pdhD*		MnTg02_02243, MnTg02_02918, MnTg02_03098	MnTg03_01036	MnTg04_00888, MnTg04_01408
**TCA cycle**					
citrate synthase [EC:2.3.3.1]					
K01647	*cs*	MnTg01_01075	MnTg02_01493, MnTg02_02258	MnTg03_01177, MnTg03_01441	MnTg04_00801
aconitate hydratase [EC:4.2.1.3]					
K01681	*aco*	MnTg01_01050	MnTg02_02005		
isocitrate dehydrogenase [EC:1.1.1.42]					
K00031	*icd*	MnTg01_01082	MnTg02_03321	MnTg03_00115	MnTg04_01734
K00030	*idh3*				MnTg04_01169
2-oxoglutarate dehydrogenase [EC:1.2.4.2, EC:2.3.1.61, EC:1.8.1.4]					
K00164	*sucA*		MnTg02_02241	MnTg03_01203	
K00658	*sucB*		MnTg02_02242		MnTg04_00887, MnTg04_01266
K00382	*pdhD*		MnTg02_02243, MnTg02_02918, MnTg02_03098	MnTg03_01036	MnTg04_00888, MnTg04_01408
2-oxoglutarate/2-oxoacid ferredoxin oxidoreductase subunit alpha [EC:1.2.7.3 1.2.7.11]					
K00174	*korA*	MnTg01_01051	MnTg02_00935, MnTg02_02706		MnTg04_01740
K00175	*korB*		MnTg02_00934, MnTg02_02707		MnTg04_01741
succinyl-CoA synthetase [EC:6.2.1.5]					
K01902	*sucD*	MnTg01_01252	MnTg02_00186, MnTg02_02240, MnTg02_03069		
K01903	*sucC*	MnTg01_01253, MnTg01_01319	MnTg02_00187, MnTg02_02239, MnTg02_03070	MnTg03_00263	
succinate dehydrogenase [EC:1.3.5.1 1.3.5.4]					
K00239	*sdhA*	MnTg01_00270	MnTg02_02871		MnTg04_01657
K00240	*sdhB*	MnTg01_00267	MnTg02_02870		MnTg04_01658
K00241	*sdhC*	MnTg01_00269	MnTg02_02873	MnTg03_00552	MnTg04_01575
K00242	*sdhD*	MnTg01_00268	MnTg02_02872	MnTg03_00553	MnTg04_01656
fumarate hydratase [EC:4.2.1.2]					
K01679	*fumC*		MnTg02_00912	MnTg03_00279	MnTg04_00517
malate dehydrogenase [EC:1.1.1.37, EC:1.1.1.38, EC:1.1.1.40]					
K00027	*maeA*				MnTg04_01330
K00028	*maeB*		MnTg02_01470	MnTg03_01103	
K00024	*mdh*	MnTg01_00090, MnTg01_01094	MnTg02_02238		MnTg04_00628
**Fumarate reductoin**					
fumarate reductase [EC:1.3.5.4]					
K00244	*frdA*		MnTg02_01694		
K00245	*frdB*		MnTg02_01697		
K00246	*frdC*		MnTg02_01696		
K00247	*frdD*		MnTg02_01695		
**Pentose phosphate pathway (Pentose phosphate cycle)**					
ribulose-phosphate 3-epimerase [EC:5.1.3.1]					
K01783	*rpe*		MnTg02_00609, MnTg02_01674		MnTg04_00864
ribose 5-phosphate isomerase A [EC:5.3.1.6]					
K01807	*rpiA*		MnTg02_03138	MnTg03_01440	MnTg04_01742
transketolase [EC:2.2.1.1]					
K00615	*tkt*	MnTg01_01286, MnTg01_01287	MnTg02_00607, MnTg02_01678		MnTg04_00391
transaldolase [EC:2.2.1.2]					

K00616	*tal*	MnTg01_00894	MnTg02_02246	MnTg03_01128	
**PRPP biosynthesis, ribose 5P => PRPP**					
ribose-phosphate pyrophosphokinase [EC:2.7.6.1]					
K00948	*prps*		MnTg02_02179	MnTg03_01076	MnTg04_00711
**Glycoside Hydrolase family**					
GH3			MnTg02_00866	MnTg03_00791	
GH15			MnTg02_00960		
GH23			MnTg02_01257, MnTg02_01649	MnTg03_00026	
GH103			MnTg02_01527, MnTg02_02029, MnTg02_02454, MnTg02_02455, MnTg02_03371	MnTg03_01580	MnTg04_01175
GH130			MnTg02_00065	MnTg03_00169, MnTg03_01566	
**Beta oxidation**					
acyl-CoA dehydrogenase [EC:1.3.8.7; EC:1.3.99.-]					
K00249	*acd*		MnTg02_00525		MnTg04_00612
K06445	*fadE*		MnTg02_00998	MnTg03_00798	MnTg04_00033, MnTg04_00629, MnTg04_01365
enoyl-CoA hydratase [EC:4.2.1.17]					
K01692	*echA*		MnTg02_01306, MnTg02_01755, MnTg02_03158	MnTg03_01188	
3-hydroxyacyl-CoA dehydrogenase / enoyl-CoA hydratase / 3-hydroxybutyryl-CoA epimerase [EC:1.1.1.35 4.2.1.17 5.1.2.3]					
K01782	*fadJ*			MnTg03_00787	MnTg04_01172
**Energy metabolism**					
**NADH:quinone oxidoreductase (Nuo)**					
NADH-quinone oxidoreductase subunit A [EC:1.6.5.3]					
K00330	*nuoA*	MnTg01_00470, MnTg01_00575	MnTg02_02695	MnTg03_01238	MnTg04_00563
K00331	*nuoB*	MnTg01_00471, MnTg01_00576	MnTg02_02694	MnTg03_01239	MnTg04_00564
K00332	*nuoC*		MnTg02_02693	MnTg03_01240	MnTg04_00565
K00333	*nuoD*	MnTg01_00716	MnTg02_03498	MnTg03_01241	MnTg04_00100
K00334	*nuoE*		MnTg02_03497	MnTg03_01242	MnTg04_00099
K00335	*nuoF*		MnTg02_03496	MnTg03_01295	MnTg04_00098
K00336	*nuoG*		MnTg02_03494		MnTg04_01527
K00337	*nuoH*	MnTg01_00717	MnTg02_03493		MnTg04_01526
K00338	*nuoI*	MnTg01_00718	MnTg02_01613, MnTg02_03492		MnTg04_01525
K00339	*nuoJ*		MnTg02_03491		
K00340	*nuoK*	MnTg01_00720	MnTg02_03490		
K00341	*nuoL*	MnTg01_00205	MnTg02_03489		MnTg04_00651
K00342	*nuoM*		MnTg02_03488	MnTg03_01475	MnTg04_00652
K00343	*nuoN*	MnTg01_00204	MnTg02_03487	MnTg03_01474	MnTg04_00653
**Cytochrocomplex respiratory unit (III, or Complex III)**					
ubiquinol-cytochrome c reductase cytochrome b subunit					
K00412	*cytB/petB*	MnTg01_00116	MnTg02_00357	MnTg03_01340	MnTg04_00831
K00413	*cyt1/petC*			MnTg03_00836	MnTg04_00830
K00411	*petA*		MnTg02_00356	MnTg03_01339	MnTg04_00832
**aa3-type cytochrome c oxidase (aa3)**					
cytochrome c oxidase [EC:1.9.3.1]					
K02275	*coxB*	MnTg01_00145	MnTg02_00520, MnTg02_01049, MnTg02_01539	MnTg03_01559	MnTg04_00849, MnTg04_01709

K02274	*coxA*	MnTg01_00146	MnTg02_00744, MnTg02_01540, MnTg02_02111, MnTg02_02977		MnTg04_00850, MnTg04_01710
K02276	*coxC*		MnTg02_01544, MnTg02_02112, MnTg02_02113	MnTg03_01563	MnTg04_00852
K02258	*cox11*		MnTg02_01543		MnTg04_00851
K02259	*cox15*	MnTg01_00148	MnTg02_01427	MnTg03_00109	MnTg04_00856
**bd-type cytochrome d ubiquinol oxidase (bd)**					
cytochrome d ubiquinol oxidase subunit I [EC:1.10.3.14]					
K00425	*cydA*		MnTg02_02023		
K00426	*cydB*		MnTg02_02021, MnTg02_02022		
**cbb3-type cytochrome c oxidase (cbb3)**					
cytochrome c oxidase cbb3-type subunit I [EC:1.9.3.1]					
K00404	*ccoN*		MnTg02_03088		
K00405	*ccoO*		MnTg02_03089		
K00407	*ccoQ*		MnTg02_03090		
K00406	*ccoP*		MnTg02_03091		
**F-type ATPase (V, or Complex V)**					
F-type H+-transporting ATPase subunit alpha [EC:3.6.3.14]					
K02111	*atpA*			MnTg03_00484	MnTg04_01419
K02112	*atpD*		MnTg02_01632	MnTg03_00486	MnTg04_01417
K02113	*atpH*		MnTg02_02991	MnTg03_00483	MnTg04_01420
K02114	*atpC*		MnTg02_01633	MnTg03_00487	MnTg04_01416
K02115	*atpG*			MnTg03_00485	MnTg04_01418
K02108	*atpB*		MnTg02_02902		MnTg04_01423
K02109	*atpF*		MnTg02_02899, MnTg02_02900	MnTg03_00482	MnTg04_01421
K02110	*atpE*		MnTg02_02901	MnTg03_00481	MnTg04_01422
V/A-type ATPase subunit alpha [EC:3.6.3.14]					
K02119	*atpC*	MnTg01_00578			
K02122	*atpF*	MnTg01_00122			
K02107	*atpH*	MnTg01_00018			
**Chemotaxis**					
**Chemotaxis**					
chemotaxis protein					
K03407	*cheA*	MnTg01_00327	MnTg02_02415		
K03412	*cheB*	MnTg01_00328	MnTg02_02412		
K03410	*cheC*	MnTg01_00326			
K00575	*cheR*	MnTg01_00325	MnTg02_02411		
K03408	*cheW*	MnTg01_00329	MnTg02_02414		
K03413	*cheY*	MnTg01_00060, MnTg01_00323, MnTg01_00797	MnTg02_00117, MnTg02_01020, MnTg02_02413		
K03414	*cheZ*		MnTg02_00118, MnTg02_01642		
K00337	*mcp*	MnTg01_00717	MnTg02_03493		MnTg04_01526
K02556	*motA*		MnTg02_02450		
K02557	*motB*		MnTg02_02449, MnTg02_03042	MnTg03_01155	
**Flagellar**					
bacterial flagellar assembly					
K02400	*flhA*		MnTg02_03430		
K02401	*flhB*		MnTg02_01163		
K02402	*flhC*		MnTg04_00242		
K02411	*fliH*		MnTg02_00381		
K02412	*fliI*		MnTg02_03424		
K02420	*fliQ*		MnTg02_01165		

K02419	*fliP*		MnTg02_01170		
K02421	*fliR*		MnTg02_01164		
K02410	*fliG*		MnTg02_00382		
K02416	*fliM*		MnTg02_02860		
K02417	*fliN*		MnTg02_00380, MnTg02_02078		
K02409	*fliF*		MnTg02_00383		
K02408	*fliE*		MnTg02_01166		
K02387	*flgB*		MnTg02_01168		
K02388	*flgC*		MnTg02_01167		
K02394	*flgI*		MnTg02_00169		
K02393	*flgH*		MnTg02_02855		
K02391	*flgF*		MnTg02_02858		
K02392	*flgG*		MnTg02_02857		
K02390	*flgE*		MnTg02_00177		
K02389	*flgD*		MnTg02_00385		
K02396	*flgK*		MnTg02_00176		
K02406	*fliC*		MnTg02_00002		
K02386	*flgA*		MnTg02_02856		
archaeal flagellar assembly					
K07325	*flaB*	MnTg01_00317, MnTg01_00795			
K07330	*flaG*	MnTg01_00315			
K07331	*flaH*	MnTg01_00314			
K07332	*flaI*	MnTg01_00332, MnTg01_01130			
K07333	*flaJ*	MnTg01_00331, MnTg01_01128, MnTg01_01129			
K07991	*flaK*	MnTg01_01294			
**Pilus**					
bacterial pilus					
K02651	*pilA*		MnTg02_01021, MnTg02_01055, MnTg02_01255		
K02652	*pilB*				MnTg04_00433
K02653	*pilC*				MnTg04_00432
K02654	*pilD*		MnTg02_02525	MnTg03_00444	MnTg04_00431
K02655	*pilE*				MnTg04_00166
K02656	*pilF*			MnTg03_01043	
K02657	*pilG*			MnTg03_00258	MnTg04_00982
K02658	*pilH*			MnTg03_00259	MnTg04_00981
K02659	*pilI*			MnTg03_00260	MnTg04_00980
K02660	*pilJ*				MnTg04_00979
K02666	*pilQ*				MnTg04_01651
K02665	*pilP*		MnTg02_01930	MnTg03_00375	MnTg04_01652
K02664	*pilO*		MnTg02_01929	MnTg03_00374	MnTg04_01653
K02663	*pilN*		MnTg02_01928	MnTg03_00373	MnTg04_01654
K02662	*pilM*		MnTg02_01927	MnTg03_00372	MnTg04_01655
K02669	*pilT*			MnTg03_00156	MnTg04_00441, MnTg04_00442, MnTg04_01045
K02670	*pilU*			MnTg03_00155	
K02671	*pilV*				MnTg04_00096, MnTg04_00732
K02672	*pilW*				MnTg04_00095, MnTg04_00731
K02674	*pilY1*				MnTg04_00167
K02676	*pilZ*			MnTg03_00786	MnTg04_01223
K02487	*pilL*				MnTg04_01234
