# Supplementary material for: Microbial metabolisms in an abyssal ferromanganese crust from the Takuyo-Daigo Seamount as revealed by metagenomics
Source: PLoS One. 2019 Nov 8;14(11):e0224888. doi: 10.1371/journal.pone.0224888 (PMC6839870; doi:10.1371/journal.pone.0224888)
Supplement: S2 Table — (PDF) [file pone.0224888.s008.pdf]

S2 Table. List of accession numbers used in Fig.1

Accession number (IMG or NCBI)	Species name
2504643023	*Caldiarchaeum subterraneum*
2510065023	*Nitrososphaera gargensis*
2757320681	*Nitrosotalea devanaterra*
2630968650	*Nitrosotenuis cloacae*
2579779048	*Nitrosotenuis chungbukensis*
2630968793	*Nitrosopelagicus brevis*
641522613	*Cenarchaeum symbiosum*
2537561915	*Nitrosoarchaeum limnia*
651324076	*Nitrosoarchaeum koreensis*
2757320762	*Nitrosomarinus catalina*
2627853696	*Nitrosopumilus piranensis*
641228499	*Nitrosopumilus maritimus*
639857004	*Mariprofundus ferrooxydans*
639633036	*Magnetococcus marinus*
637000058	*Pelagibacter ubique*
2517287015	*Sphingomonas melonis*
641228493	*Gluconacetobacter diazotrophicus*
637000241	*Rhodospirillum rubrum*
639633056	*Roseobacter denitrificans*
637000135	*Hyphomonas neptunium*
640753040	*Parvibaculum lavamentivorans*
2524614806	*Cucumibacter marinus*
637000038	*Bradyrhizobium japonicum*
2521172615	*Kaistia granuli*
644736401	*Rhizobium leguminosarum*
637000159	*Mesorhizobium loti*
649633090	*Rhodomicrobium vannielii*
648028034	*Hyphomicrobium denitrificans*
2521172694	*Hyphomicrobium zavarzinii*
2786546836	*Methyloligella halotolerans*
2630968843	*Methyloceanibacter caenitepidi*
642555101	*Acidithiobacillus ferrooxidans*
2523533554	*Thermithiobacillus tepidarius*
646311935	*Halothiobacillus neapolitanus*
2506520043	*Thioalkalivibrio thiocyanoxidans*
2526164694	*Sinobacter flavus*
637000343	*Xanthomonas campestris*
2506520000	*Stenotrophomonas maltophila*
638341135	*Nitrococcus mobilis*
2751185661	*Woeseia oceani*
2721755797	*Steroidobacter denitrificans*
637000166	*Methylococcus capsulatus*
2517287033	*Methylobacter luteus*
2747842540	*Granulosicoccus antarcticus*
2512564004	*Methylophaga nitratireducens*
2508501047	*Beggiatoa alba*
2540341246	*Thiomicrospira kuenenii*
637000222	*Pseudomonas putida*
2524614562	*Halomonas anticariensis*
2521172684	*Alteromonas macleodii*
637000258	*Shewanella oneidensis*
637000107	*Escherichia coli*
646862349	*Vibrio cholera*
2527291500	SCGC AAA007-O23
2630968322	SCGC AAA799-P11
2708742729	RBG_16_64_48
2654587955	WOR_SG8_31
2651869885	bin 20_j1
2651869504	SAG 1868_B
LXTF01000000	SPGG7
LXTL01000000	SPGG1
